# Impact of vaccination on kinetics of neutralizing antibodies against SARS-CoV-2 by serum live neutralization test based on a prospective cohort

**DOI:** 10.1080/22221751.2022.2146535

**Published:** 2023-01-19

**Authors:** Liguo Zhu, Naiying Mao, Changhua Yi, Aidibai Simayi, Jialu Feng, Yi Feng, Min He, Songning Ding, Yin Wang, Yan Wang, Mingwei Wei, Jie Hong, Chuchu Li, Hua Tian, Lu Zhou, Jiefu Peng, Shihan Zhang, Ci Song, Hui Jin, Fengcai Zhu, Wenbo Xu, Jun Zhao, Changjun Bao

**Affiliations:** a NHC Key Laboratory of Enteric Pathogenic Microbiology, Jiangsu Provincial Center for Disease Control and Prevention, Nanjing, China; b National Institute for Viral Disease Control and Prevention, Chinese Center for Disease Control and Prevention, Beijing, China; c Nanjing Infectious Diseases Clinical Medical Center (The Second Hospital of Nanjing, Nanjing University of Chinese Medicine), Nanjing, P.R China; d Department of Epidemiology and Health Statistics, School of Public Health, Southeast University, Nanjing, China; e School of Public Health, Nanjing Medical University, Nanjing, People’s Republic of China; f Nanjing Municipal Center for Disease Control and Prevention, Nanjing, People’s Republic of China; g Yangzhou Center for Disease Control and Prevention, Yangzhou, Pople's Republic of China; h The Third People's Hospital of Yangzhou, Yangzhou, People’s Republic of China

**Keywords:** SARS-CoV-2, Delta variant, CoronaVac, breakthrough infection, neutralizing antibody

## Abstract

How much the vaccine contributes to the induction and development of neutralizing antibodies (NAbs) of breakthrough cases relative to those unvaccinated-infected cases is not fully understood. We conducted a prospective cohort study and collected serum samples from 576 individuals who were diagnosed with SARS-CoV-2 Delta strain infection, including 245 breakthrough cases and 331 unvaccinated-infected cases. NAbs were analysed by live virus microneutralization test and transformation of NAb titre. NAbs titres against SARS-CoV-2 ancestral and Delta variant in breakthrough cases were 7.8-fold and 4.0-fold higher than in unvaccinated-infected cases, respectively. NAbs titres in breakthrough cases peaked at the second week after onset/infection. However, the NAbs titres in the unvaccinated-infected cases reached their highest levels during the third week. Compared to those with higher levels of NAbs, those with lower levels of NAbs had no difference in viral clearance duration time (*P*>0.05), did exhibit higher viral load at the beginning of infection/maximum viral load of infection. NAb levels were statistically higher in the moderate cases than in the mild cases (*P*<0.0001). Notably, in breakthrough cases, NAb levels were highest longer than 4 months after vaccination (Delta strain: 53,118.2 U/mL), and lowest in breakthrough cases shorter than 1 month (Delta strain: 7551.2 U/mL). Cross-neutralization against the ancestral strain and the current circulating isolate (Omicron BA.5) was significantly lower than against the Delta variant in both breakthrough cases and unvaccinated-infected cases. Our study demonstrated that vaccination could induce immune responses more rapidly and greater which could be effective in controlling SARS-CoV-2.

## Introduction

Evidences since the global pandemic of coronavirus disease 2019 (COVID-19) suggest that it could be controlled with effective vaccines and the non-pharmaceutical interventions (NPI) strategy. Since their launch in 2021 in China, two kinds of domestic vaccines (BBIBP and CoronaVac, containing antigen from the ancestral SARS-CoV-2 strain) [1] obtained Emergency Use Listing by WHO and have been effective in preventing COVID-19 caused by severe acute respiratory syndrome coronavirus 2 (SARS-CoV-2). In the phase 3 trial [2], interim results of CoronaVac had been shown to prevent symptomatic COVID-19 (83.5% relative to placebo) and COVID-19-related hospitalization (100%) after full vaccination (two doses). A prospective national cohort in Chile [3] showed the adjusted vaccine effectiveness (CoronaVac) for the prevention of COVID-19, hospitalization, and ICU admission were 65.9%, 87.5%, and 90.3%, respectively. BBIBP-CorV showed similar effectiveness with BNT162b2 against COVID-19-related hospitalizations from the Delta (B.1.617.2, first detected in India) variant [4]. In addition, SARS-CoV-2 mRNA vaccines and adenovirus vectored vaccine (Ad26.COV2.S) have been remarkably successful in inducing neutralizing humoral and cellular immunity [5–6]. Furthermore, the number and depth of experimental studies linking the clinical outcomes and quantification of the neutralizing antibodies (NAbs), T cell responses, and various cytokine levels in vaccinated/non-vaccinated subjects are unprecedented in the history of human vaccination [7-8].

However, breakthrough infections have emerged in vaccine recipients, as reported in many countries. The breakthrough infections in fully vaccinated individuals are increasing gradually, which suggested that present SARS-CoV-2 vaccines do not offer enduring immunity, especially against variants with immune escape ability [9]. All commercial vaccines up to 2021 were based on the S1-RBD sequences of the original Wuhan-1 reference strain. Thus, the obvious limitation of these vaccines is ineffective in blocking of viral entry by VOCs carrying a.a. mutations in the ACE-2 RBD. The occurrence of breakthrough infections has been associated with the decline of NAbs after vaccination [10,11]. Besides, the highly transmissible Delta variant shows the potential ability of immune escape and the ability to evade vaccines [12,13]. Compared to NAbs levels induced by infection, vaccination may induce similar or lower NAbs levels that decay faster [14]. How much the vaccine contributes to the induction and development of NAbs of breakthrough cases relative to those unvaccinated-infected cases is not fully understood.

On 20 July 2021, Nanjing notified that 9 of the regular nucleic acid test samples of Lukou International Airport staff were positive which then caused a large cluster of COVID-19 outbreaks in Nanjing and Yangzhou, Jiangsu Province, China. We conducted this study to explore the impact of vaccine by comparing the kinetics of NAb between unvaccinated-infected cases and breakthrough cases and understand how the vaccine contributes to the induction and development of NAbs, especially in the context of evolving variants.

## Results

### Demographic characteristics and immunization history of the participants

**Figure 1.**
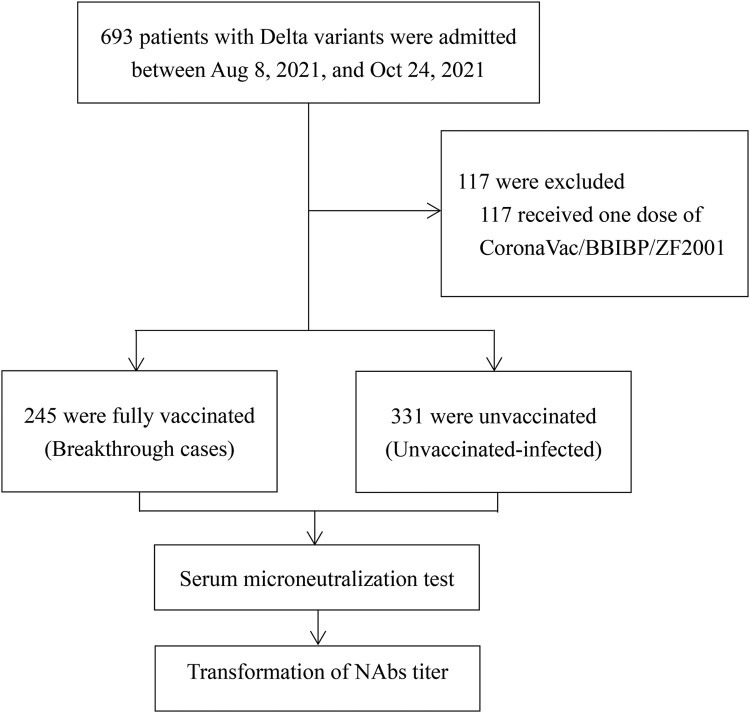
Study Enrolment and Outcomes (8 August 2021–24 October 2021). Note: NAbs denotes neutralizing antibodies.

Among the 693 eligible patients enrolled during the period from 8 August 2021, to 24 October 2021, a total of 1432 samples from 576 patients were obtained (Figure 1). Among them, 660 sera samples from 245 patients were collected with full SARS-CoV-2 vaccine immunization, 772 sera samples from 331 cases without history of SARS-CoV-2 vaccine immunization. Among breakthrough cases, the mean (SD) age was 46.7 (11.7) years old and 58 (23.6%) cases were male (Table 1). Whereas, the mean (SD) age of unvaccinated-infected cases was 53.7 (16.0) years old including 127 (38.3%) males and 204 (61.6%) females. In breakthrough infectious patients, the sampling time was 3–46 days after the date of onset/infection while unvaccinated infections were 3–48 days. The time between the second dose of vaccine and days of onset/infection was 15–197 days in breakthrough cases.

**Table 1.** Demographic characteristics and Vaccination history of the participants.

	Total participants (*N *= 576)	Breakthrough cases (*n *= 245)	Unvaccinated-infected cases (*n *= 331)	*P*
Age (Mean±SD)	50.7±18.4	46.7±11.7	53.7±16.0	<0.0001
Gender (n[%of total])				<0.0001
Female	391(67.9%)	187(76.3%)	204(61.6%)	
Male	185(32.1%)	58(23.6%)	127(38.3%)	
Vaccines (n[%of total])				---
BBIBP	43(7.5%)	43(17.6%)	---	
CoronaVac	156(27.1%)	156(63.7%)	---	
ZF2001	16(2.8%)	16(6.5%)	---	** **
BBIBP&CoronaVac	30(5.2%)	30(12.2%)	---	
Clinical classification (n[%of total])				<0.0001
Mild	112(19.4%)	42(17.1%)	70(21.1%)	
Moderate	419(72.7%)	203(82.9%)	216(65.3%)	
Severe/Critical	43(7.5%)	0(0%)	43(13.0%)	** **
Untyped	2(0.3%)	0(0%)	2(0.6%)	

All patients who received injections were vaccinated with BBIBP (Inactivated COVID-19 Vaccine manufactured by Beijing Institute of Biological Products Co., Ltd.), CoronaVac (Inactivated COVID-19 Vaccine manufactured by Sinovac Life Sciences Co., Ltd), and ZF2001 (adjuvanted protein subunit COVID-19 vaccine manufactured by Anhui Zhifei Longcom) or mixed inoculation. Among 245 breakthrough infections patients, 43 (17.6%), 156 (63.7%), and 16 (6.5%) patients were fully vaccinated with BBIBP, CoronaVac, and ZF2001, respectively. And 30 (12.2%) had heterologous prime series with BBIBP and CoronaVac.

According to *Diagnosis and Treatment Protocol for Novel Coronavirus Pneumonia* [15], the clinical spectrum of COVID-19 ranges from mild to critical cases. Among those patients with breakthrough infections, the most common cases were moderate cases 82.9% (203/245), and mild cases were reported by 17.1% (42/245). While, among those unvaccinated-infected patients, the most common cases were also moderate cases 65.3% (216/331) in proportion, mild cases were 21.1% (70/331), severe cases 13% (43/331) and untyped 0.6% (2/331). The clinical classification of breakthrough infection and unvaccinated-infected cases was mainly the moderate type. Unvaccinated-infected cases had a higher rate of severe illness. The distribution of clinical classification between breakthrough cases and unvaccinated-infected cases showed a significant statistical difference (*χ^2 ^*= 51.65, *P*<0.0001).

### Kinetics of NAb levels over time after onset/infection

**Figure 2.**
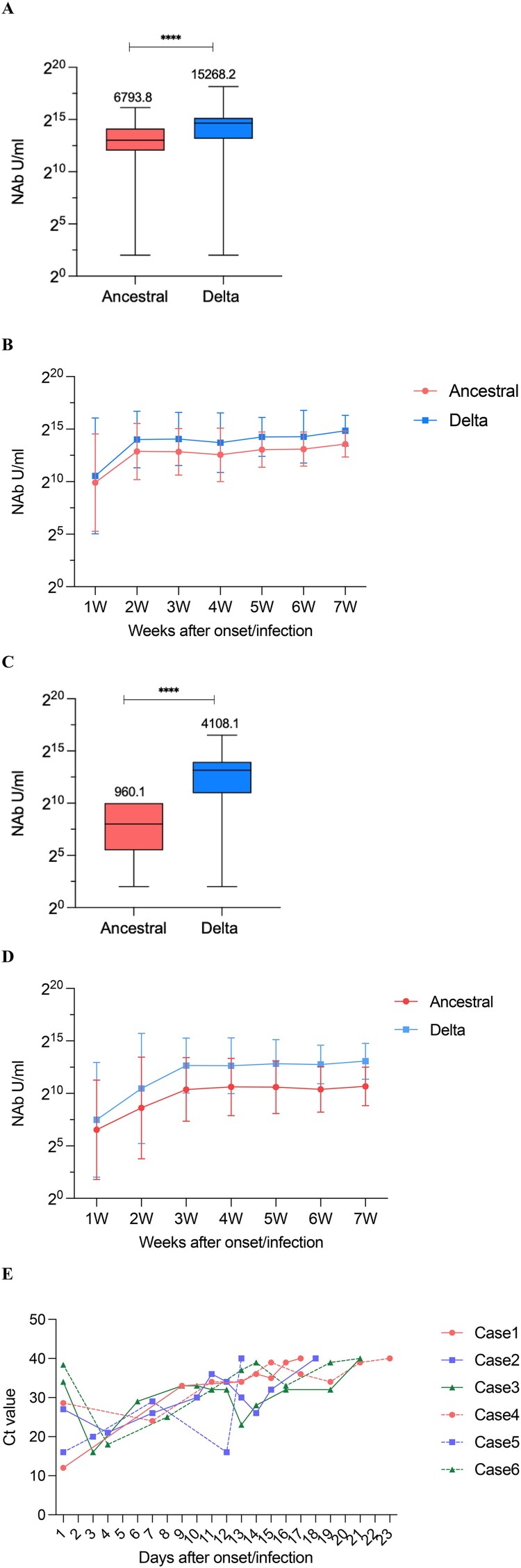
Trends in NAb levels over time from 1 to 7 weeks of the onset/infection. (A) NAbs of the ancestral and Delta strains in breakthrough cases. (B) Neutralizing curves of the ancestral and Delta strains in breakthrough cases from 1 to 7 weeks of the onset/infection by live virus microneutralization assay. (C) NAbs of the ancestral and Delta strains in unvaccinated-infected cases. (D) Neutralizing curves of the ancestral and Delta strains in unvaccinated-infected cases from 1 to 7 weeks of the onset/infection by live virus microneutralization assay. ****P < 0.0001. (E) Trends of Ct value among different NAbs value over days after symptom onset/infection. Case1-3: breakthrough cases with high-medium-low NAb level, case4-6: unvaccinated-infected cases with high-medium-low NAb level.

We investigated 7-week serum NAbs titres after symptom onset/infection. Most cases had only one blood sample per week. Results of breakthrough cases showed that the Delta variant maintained overall higher NAbs than the ancestral strain (the geometric mean concentration [GMC] of ancestral strain: 6793.8 U/mL, Delta strain: 15,268.2 U/mL, *p*<0.0001) (Table 2) (Figure 2(A,B)); NAb levels in these two strains changed consistently after the onset/infection of the disease, and both peaked at the second week approximately (ancestral strain: 7478.1 U/mL; Delta strain: 16,511.1 U/mL). Afterwards, the increased levels gradually stabilized. Unvaccinated-infected cases began to increase rapidly within three weeks after onset/infection (ancestral strain: 1335.1 U/mL; Delta strain: 6476.5 U/mL), and NAb levels showed a slow and continuous upward trend after the third week. Similarly, the level of NAbs against the Delta strain was higher than in the ancestral strain for the cases without immunization history (ancestral strain: 960.1 U/mL; Delta strain: 4108.1 U/mL). For both strains, NAbs titres in breakthrough cases were higher than those in unvaccinated-infected cases, and plateaued earlier (Figure 2(C,D)). The concentration ratio of breakthrough cases to unvaccinated-infected cases became progressively smaller as the time of onset/infection continued for both ancestral and Delta strains. The immune responses between inactivated vaccine and subunit vaccine recipients in breakthrough infections did not show statistical differences (Ancestral: *p* = 0.327, Delta: *p* = 0.330).

**Table 2.** The geometric mean concentrations (U/mL) of the NAb against ancestral and Delta strains in breakthrough cases and unvaccinated-infected cases from 1–7 weeks after the onset/infection.

		Overall	1W	2W	3W	4W	5W	6W	7W
Breakthrough cases	N	660	21	108	193	177	116	37	8
	Ancestral	6793.8	964.2	7478.1	6843.6	5495.6	7489.4	6317.4	8304.5
	Delta	15268.2	1546.4	16511.1	16095.3	11620.5	17423.2	14612.5	19598.4
Unvaccinated-infected cases	N	772	12	227	87	136	156	114	40
	Ancestral	960.1	93.1	393.3	1335.1	1571.9	1557.9	1338.9	1636.1
	Delta	4108.1	179.9	1422.5	6476.5	6388.1	7294.9	6923.2	8594.2
*P* value of breakthrough cases VS. Unvaccinated-infected cases	Ancestral	<0.0001	0.053	<0.0001	<0.0001	<0.0001	<0.0001	<0.0001	<0.0001
	Delta	<0.0001	0.142	<0.0001	<0.0001	<0.0001	<0.0001	<0.0001	0.001
GMC ratio of breakthrough/unvaccinated-infected	Ancestral	7.1	10.4	17.3	5.1	3.5	4.8	4.7	5.1
	Delta	3.7	8.6	10.7	2.5	1.8	2.4	2.1	2.3

Note: NAb denotes neutralizing antibody, GMC denotes geometric mean concentration, and W denotes week. Kruskal Wallis Test was used for the comparison of NAbs in the two groups.

### Kinetics of PCR Ct value according to NAb levels

To infer the relationship between NAbs titres and viral clearance, we selected 38 cases (18 breakthrough cases and 20 unvaccinated-infected cases) with continuous PCR cycle threshold (Ct) values throughout the course of infection and divided them into three groups according to the NAb levels against Delta strain (high group: NAb ≥ Thirdquartile, medium group: Thirdquartile < NAb< Firstquartile, low group: NAb≤ Firstquartile). Further, the duration time from onset/infection to virus clearance, the Ct difference value between the maximum and minimum Ct values, and the ratio of the Ct difference value to the virus clearance duration time of these three groups were analysed (Table 3). Compared to those with higher levels of NAbs, those with lower levels of NAbs had no difference in viral clearance duration time (*P*>0.05), but did reveal both higher viral load at the beginning of infection and maximum viral load of infection, thus showing greater variation in their Ct values per day, implying a faster rate of cleaning virus during the similar viral clearance course. Intuitively, six representative cases chosen to depict their Ct values trend concerning post-onset/infection times are exhibited in Figure 2(E).

**Table 3.** Viral clearance time and Ct value differences in different neutralizing antibody levels.

	Groups accortding to NAb titre (Median [IQR])			* *
	High	Medium	Low	*p* value
Breakthrough cases				
Viral clearance time (days)	19[16-23.25]	17.5[14-20.25]	17.5[11.5-19.75]	0.454
Ct value _Max-Min_	13[9.5-18]	19.5[14.5-21.5]	22[18.5-24.75]	0.047
Ct value _Max-Min_/Viral clearance time	0.69[0.45-1.07]	1.11[0.74-1.55]	1.38[1.01-1.89]	0.033
Unvaccinated-infected cases				
Viral clearance time (days)	18[12-24.25]	11.5[9.5-15]	13[10.75-18.25]	0.267
Ct value _Max-Min_	19.5[14.75-26.25]	21.5[15.25-24]	20.5[19-23.75]	0.929
Ct value _Max-Min_/Viral clearance time	1.26[0.61-3.02]	1.64[1.21-2.50]	1.73[1.08-2]	0.605

Note: Ct denotes cycle threshold.

### Kinetics of NAbs according to clinical status

**Figure 3.**
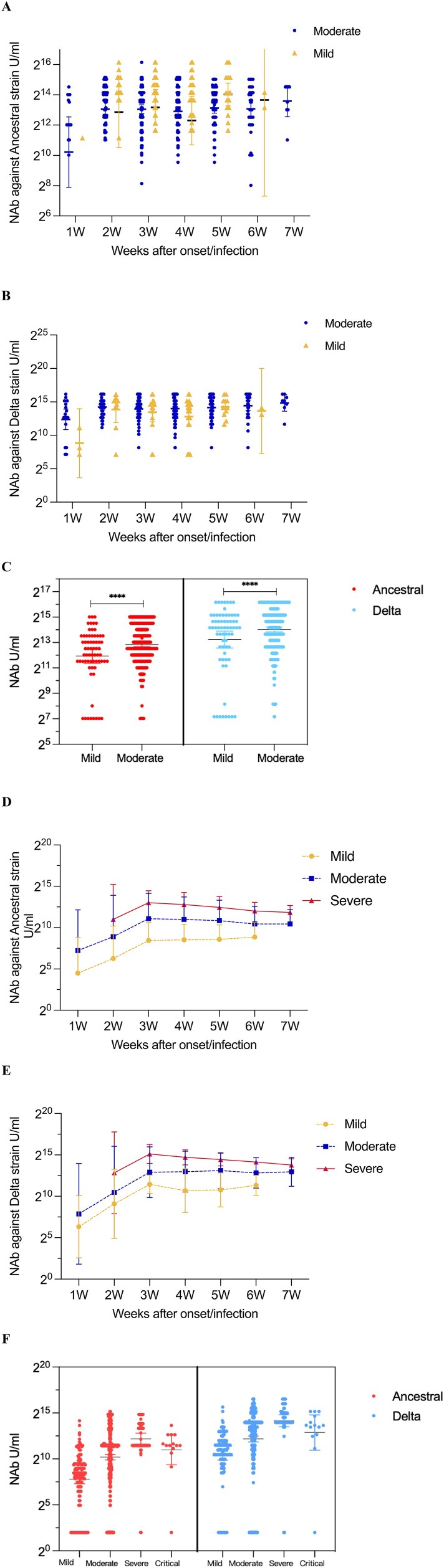
Kinetics of NAb according to clinical status. In breakthrough cases (A-C): (A) NAbs of the ancestral strain from 1-7 weeks of the onset/infection by live virus microneutralization assay. (B) NAbs of the Delta strain from 1-7 weeks of the onset/infection by live virus microneutralization assay. (C) NAbs of the ancestral and Delta strain. In un-vaccinated-infected cases (D-E): (D) NAbs of the ancestral strain from 1-7 weeks of the onset/infection by live virus microneutralization assay. (E) NAbs of the Delta strain from 1-7 weeks of the onset/infection by live virus microneutralization assay. (F) NAbs of the ancestral and Delta strain. Each circle represents the titer for a serum sample. ****P < 0.0001.

In breakthrough cases, NAb levels of moderate cases against both ancestral and Delta strains were all significantly higher than those in the mild cases. Specifically, the GMC of moderate cases and mild cases against ancestral strain were 7873.6 and 2908.4 U/mL (*p*<0.0001), respectively (Figure 3(A,C)). While, the GMC of moderate cases and mild cases against Delta strain were 17,487.4 and 6971.0 U/mL, respectively (Figure 3(B,C)). In both ancestral and Delta strains, NAb levels of moderate cases significantly increased in the second week after onset/infection (ancestral strain: 8482.3 U/mL, Delta strain: 18,653.9 U/mL), and then showed a steady trend. For mild cases, NAb levels also significantly increased in the second week after onset/infection in both strains (ancestral strain: 3431.3 U/mL, Delta strain: 7420.3 U/mL), but showed a significant decrease until the fourth week after onset/infection (ancestral strain: 1635.6 U/mL, Delta strain: 5053.8 U/mL).

In those unvaccinated infections, NAb levels of the ancestral strain and Delta strain had the same trend with disease severity. Severe cases had the highest levels (ancestral strain: 4663.3 U/mL, Delta strain: 18,270.4 U/mL) while mild cases had the lowest levels (ancestral strain: 219.5 U/mL, Delta strain: 1309.1 U/mL). For both ancestral and Delta strains, antibody levels peaked at the third week after the onset/infection for mild cases (ancestral: 347.9 U/mL, Delta: 2794.1 U/mL), moderate cases (ancestral: 2165.9 U/mL, Delta: 7697.0 U/mL), and severe cases (ancestral: 8235.1 U/mL, Delta: 35,517.9 U/mL), respectively (Figure 3(D–F)).

### Impact of the duration of SARS-CoV-2 vaccination on NAbs over the time after onset/infection in breakthrough cases

**Figure 4.**
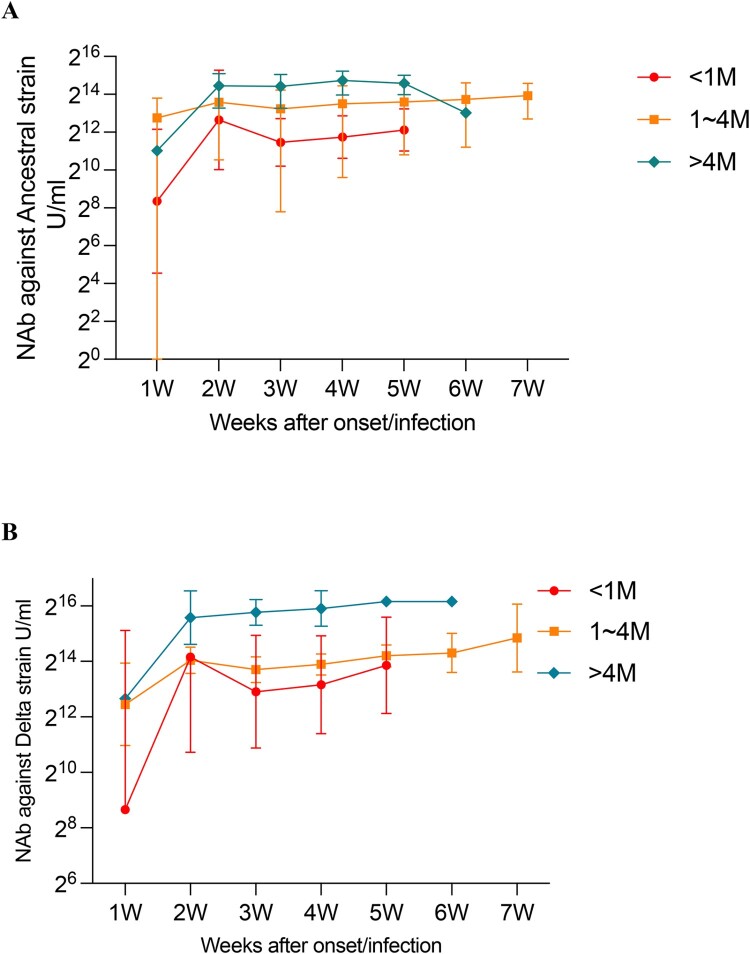
Impact of duration after SARS-CoV-2 vaccination on NAbs against ancestral and Delta variants in breakthrough cases. (A) Effect of SARS-CoV-2 vaccination duration on NAbs against the ancestral strain over weeks after onset/infection. (B) Effect of SARS-CoV-2 vaccination duration on NAbs against the Delta strain over weeks after onset/infection.

We analysed the NAbs against ancestral and Delta according to both the duration of SARS-CoV-2 vaccination and the time after onset/infection in breakthrough cases. The results showed that at the same post-onset/infection time point, the longer the duration from vaccination to onset/infection, the higher level of NAbs produced. For both strains, NAb levels were the highest in cases longer than 4 months after vaccination (ancestral strain: 17,764.3 U/mL, Delta strain: 53,118.2 U/mL), and lowest in cases shorter than 1 month (ancestral strain: 2906.2 U/mL, Delta strain: 7551.2 U/mL) (Figure 4(A,B)). NAbs titres against SARS-CoV-2 ancestral and Delta variant in cases with time duration ≥4 months were 6.1-fold and 7.0-fold higher than those cases with time duration < 1 month, respectively (*p*<0.001). It seems that the level of NAbs was higher for those vaccinated earlier than those vaccinated later in breakthrough cases.

### Cross NAb responses against Omicron BA.5

**Figure 5.**
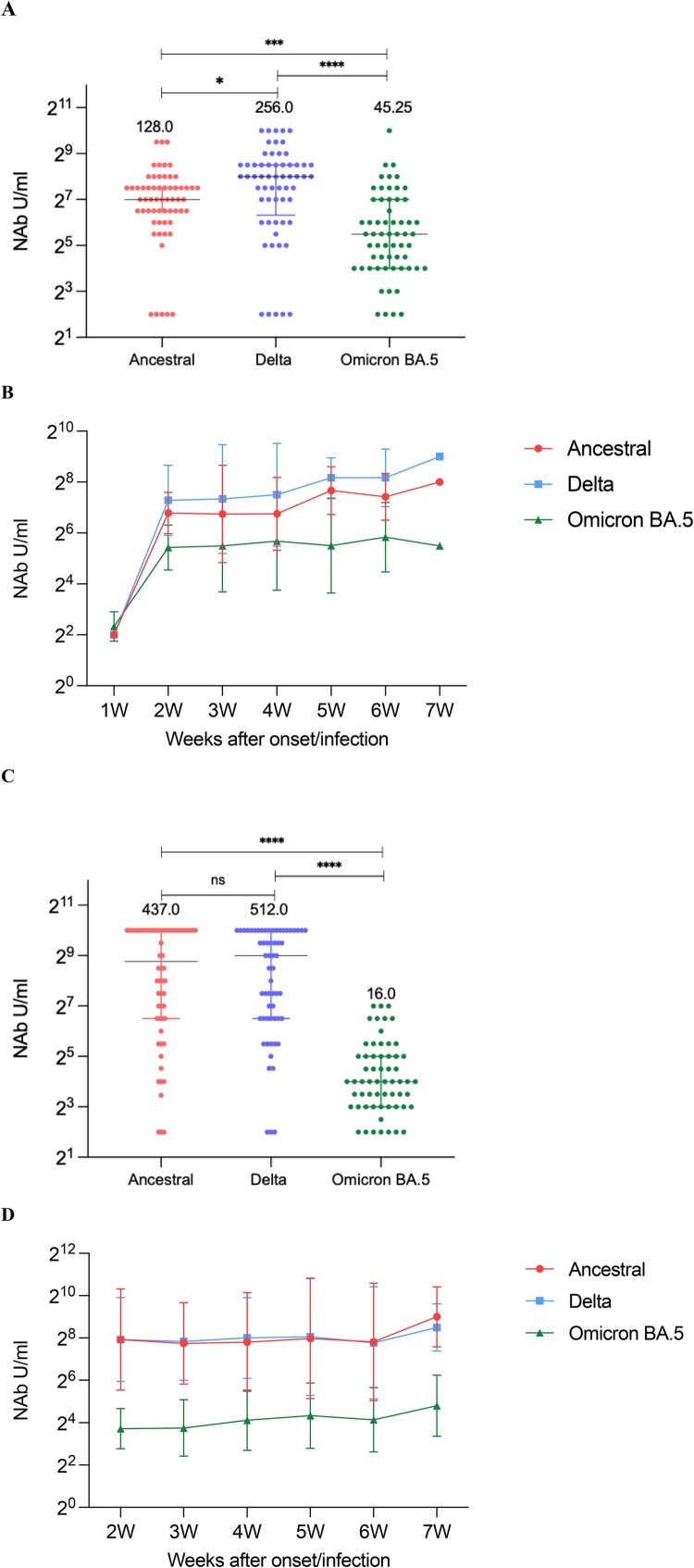
Neutralizing responses of three strains over time. (A) NAbs of the ancestral, Delta, and Omicron BA.5 in breakthrough cases. (B) Neutralizing curves of the ancestral, Delta, and Omicron BA.5 in breakthrough cases from 1 to 7 weeks of the onset/infection by live virus microneutralization assay. (C) NAbs of the ancestral, Delta, and Omicron BA.5 in unvaccinated-infected cases. Each circle represents the titre for a serum sample. (D) Neutralizing curves of the ancestral, Delta, and Omicron BA.5 in unvaccinated-infected cases from 1–7 weeks of the onset/infection by live virus microneutralization assay. ****P < 0.0001, ***P < 0.001, *P < 0.05, ns denotes no statistical differences.

We randomly selected 40 cases (120 samples) from breakthrough group and unvaccinated-infected group, then analysed cross NAbs responses and kinetics of NAbs against to three strains of SARS-CoV-2 (ancestral, Delta and Omicron BA.5). In breakthrough cases, NAb levels against Delta itself was the highest and against Omicron BA.5 was the lowest (ancestral strain: 128.0 U/mL, Delta strain: 256.0 U/mL, Omicron BA.5: 45.25 U/mL) (Figure 5(A)). Similar to those against Delta strain, NAb levels against Omicron BA.5 strain changed consistently after the onset/infection of disease, peaked at the second week, and then the increased level gradually stabilized (Figure 5(B)). In those unvaccinated-infected cases, there was no statistical difference between the NAb levels against ancestral strain and Delta strain, but the NAbs against Omicron strain was still lower (16.0 U/mL) (Figure 5(C)). Whereas, NAbs titres of Omicron showed a relatively stable trend throughout 2–7 weeks after symptom onset/infection (Figure 5(D)). Moreover, we have presented the kinetics for each individual in order to show inter-person variation in Supplementary material Figure S1A-F.

## Discussion

Individuals with SARS-CoV-2 vaccination were found to produce Nabs that inhibit the interaction between the SARS-CoV-2 receptor binding domain (RBD) and the host cell surface-expressed angiotensin-converting enzyme 2 receptor (ACE2) [16]. We separately analysed the characteristics of NAbs' response over time in breakthrough cases and unvaccinated-infected cases. The immune system of both unvaccinated infection and breakthrough infection is responding to the live replicating virus by producing binding and NAbs. Our findings show that the NAb magnitude of breakthrough cases against ancestral and Delta strains was substantially stronger than those of unvaccinated-infected cases (7.8-fold and 4.0-fold, respectively). In the present study, we found that the time from emergence to peak titre of NAbs between breakthrough cases and unvaccinated infections was different. The time breakthrough cases were in the second week after onset/infection while unvaccinated infections were in the third week. It implied that the immune response speed of breakthrough cases was faster than unvaccinated-infected cases. This may indicate that vaccination can induce robust anamnestic immune responses when the patients got SARS-CoV-2 infection [9, 17].

NAbs titres for ancestral and Delta strains maintained stable levels in both breakthrough and unvaccinated-infected cases after reaching the peak. We did not find a significant difference in the trend of decay of NAbs between the breakthrough and unvaccinated-infected cases. Both breakthrough infection and unvaccinated-infected cases remain at stable NAb levels. Estimates of the half-life of the neutralization titre after infection suggest a half-life of 55∼90 days, which may be due to lived antibody-secreting cells (which decay with a half-life of weeks) [18].

In this study, the distribution of clinical classification between breakthrough cases and unvaccinated-infected cases was different. Unvaccinated-infected cases had a higher rates of severe/critical type than those in breakthrough cases. The PCR Ct value, an alternate measure of viral load, was often taken as a measure of infectiousness [19]. It was reported that a lower PCR Ct value was related to severe COVID-19 patients [20]. Compared to those with lower levels of NAbs, those with higher levels of NAbs had no difference in viral clearance duration time, but did exhibit lower viral load at the beginning of infection/maximum viral load of infection, which would lead to a decrease in infectivity and be conducive to the prevention and control of the COVID-19 epidemic.

The concentration of NAbs in both breakthrough cases and unvaccinated infections were closely associated with the severity of the disease. The more severe the disease was, the higher the concentration of NAbs was, which was consistent with a research based on 94 COVID-19 convalescent patients in Italy [21]. Such association can be explained by the possibility that uncontrolled viral spread led to increased pathology, hyper-inflammation, and elicited overproduction of viral antigen load, which will favour humoral response [22-23]. A rhesus macaque model study indicates that the number of NAbs directed at the S protein, which mediates cellular binding, emerged as the strongest correlate of protection [24]. It may be a key protective factor to reduce the development of severe or even critically ill patients. However, estimating the protective effect of vaccination against severe illness from descriptive population-level statistics is non-trivial [25]. The present study obtained consistent findings through prospective follow-up of real-world large-scale cases, which can be used as evidence for empirical studies.

Based on microneutralization tests with large sample size, this study demonstrated that compared with the Delta variant, NAbs against the ancestral strain were significantly decreased in both breakthrough cases and unvaccinated-infected cases. We also evaluated cross-neutralization ability of 120 sera against the current circulating isolate (Omicron BA.5). The NAbs against Omicron strain was still lower than against Delta variant in both breakthrough cases and unvaccinated-infected cases. Since participants in this study were all infected with Delta variant, the neutralizing activity of Nab induced by Delta variant infection was relatively stronger against itself than other SARS CoV-2 strain. The dramatic reduction of cross neutralization might be associated with immune evasion caused by mutations in Spike protein [26-29].

Individuals who had recovered from previous SARS-CoV-2 infection and subsequently received a single BNT162b2 dose had the highest antibody levels by 10 days after vaccination [30]. A study of 180 Finnish healthcare workers indicated that vaccinees showed higher neutralization six weeks after the first dose of the COVID-19 vaccine than three weeks [31]. Unlike previous studies, we further analysed the impact of time duration after SARS-CoV-2 vaccination in breakthrough cases. Robust NAbs titres were observed in breakthrough-infected individuals with vaccination over 4 months. Our study showed that the longer time from vaccination to onset/infection, the higher level of NAbs produced. NAbs in immune breakthrough cases came from vaccination and infection induction. At the same infection time point, earlier vaccination could induce more NAbs, which means the time interval between vaccination and breakthrough infection strongly correlated with the level of antibodies against several variants [32]. The interval between vaccination and boost by natural infection is an important point, but the optimal timing of vaccinations, especially given waning responses, must take into account many considerations.

The NAb activity of this study was measured by a live virus microneutralization test, called “the gold standard” of neutralization antibody test, which can assess the ability of the serum of patients to neutralize SARS-CoV-2 comprehensively and can be used to compare the neutralization activity against different variants (ancestral strain, Delta and Omicron BA.5) and to evaluate the cross-neutralization ability.

Several limitations remain. First, we did not measure anti-S RBD IgG. It was unfortunate that the cell-based Nab assay in this work was not validated or compared with specific anti-S1-RBD IgG activity. According to the study of Venice Servellita [33] which neutralization was also measured by live virus neutralization assay, there was decreased correlation of WT spike RBD IgG antibody and neutralization with Delta (Spearman’s ρ = 0.83) and Omicron (ρ = 0.49) variants relative to WT (ρ = 0.91). It was suggesting that RBD IgG titres can be employed to predict neutralization. Secondly, in this study, the level and function of cellular immunity require further study. Thirdly, the immunity mechanism of breakthrough cases was still unknown, and it required additional study to illuminate the phenomenon of infection with the variant virus, which can be sustained with a high viral load despite high levels of NAbs to variants. Finally, we did not add a vaccination without the SARS-CoV-2 infection cohort. This made it impossible to assess the effect of SARS-CoV-2 infection on inducing NAbs in those immunized populations.

## Conclusion

Our study demonstrated that vaccination could induce immune responses more rapidly and greater that could be effective in controlling SARS-CoV-2.

## Methods

### Study design and participants

The prospective study included patients with breakthrough infection and unvaccinated infection occurred in Nanjing and Yangzhou, Jiangsu Province, China. A breakthrough infection was defined as the detection of SARS-CoV-2 on RT–PCR assay performed 14 or more days after vaccination of a second dose of CoronaVac/BBIBP/ZF2001. Unvaccinated infection was defined as patients without any SARS-CoV-2 immunization. During the period from 8 August 2021 to 24 October 2021 in Jiangsu province, China, a total of 576 patients with 1432 serum samples were collected. Those patients who had been diagnosed with SARS-CoV-2 infection confirmed by real-time PCR on nasal and/or pharyngeal swab specimens according to Diagnosis and Treatment Protocol for Novel Coronavirus Pneumonia were divided into different clinical classifications and followed up. We obtained 520 complete or near-complete viral genomes, including 167 genomes from 163 cases of Nanjing outbreak, 353 genomes form 222 cases of Yangzhou outbreak (Supplementary material Table 1). Through whole-genome sequencing analysis (Illumina, CA, USA) [34], it was found that these patients were infected with the Delta variants.

### Serum microneutralization test

Live virus MN assay was established for SARS-CoV-2 NAbs detection. Three strains of SARS-CoV-2 (Ancestral, Delta and Omicron BA.5) were used as the challenge virus, and the 50% cell culture infective dose (CCID_50_)/50μL was calculated by the formula of Kärber. Briefly, the serum samples were inactivated at 56°C for 30 min, and then diluted serially twofold, starting at dilution of 1:8 and ending at 1:1024. Fifty microliters of each diluted serum were mixed with an equal volume of challenge virus containing approximately 100 CCID_50_/50μL. After neutralization at 36.5°C for 2h in 5% CO_2_ incubator, the Vero-E6 cell suspension was added in the plate with the maintenance medium to form monolayer of cells. The plate was kept at 36.5°C for 5 days in 5% CO_2_ incubator. During this period, the cytopathic effect (CPE) was observed daily. The titre of NAbs was calculated by reed Muench method. Serum with a NAbs titre of greater than or equal to 1:8 was considered positive. In order to calculate geometric mean titre (GMT), antibody titres of < 1:8 and > 1:1024 were assigned as 1:4 and 1:1024, respectively.

### Transformation of NAbs titre

The national standard for SARS-CoV-2 NAbs (No.280034-202001) which assigned a concentration of 1000 Units per mL, available from the China National Institute for Food and Drug Control, was used to convert neutralizing titre of serum to U/mL in this study. For each assay run, the unitage constant was calculated by dividing the concentration of antibodies in the standard by the neutralizing titre of the standard in that assay run. The neutralizing titre of test serum were then multiplied by the unitage constant to obtain the transformed and comparable NAb titre in U/mL.

### Statistical analysis

Statistical analysis was performed using SPSS 26.0 (IBM SPSS Statistics) and GraphPad PRISM 9.3.1 (GraphPad Software, San Diego CA, USA). Descriptive summaries of the baseline characteristics of the participants were reported for continuous and categorical variables. For continuous variables, mean and standard deviation (SD) were reported. For categorical variables, counts and percentages of patients with positive results were summarized. We used Student’s *t*-test and Related-Samples Wilcoxon Signed Rank Test or Kruskal Wallis Test for normally and non-normally distributed variables, respectively. We compared gender and distribution of clinical stage between breakthrough cases and unvaccinated-infected cases using the chi-square test. Results that have heavily skewed distributions were normalized by log transformation. Log-transformed NAb titres against different variants were compared using Student’s T test and one-way ANOVA with Tukey’s multiple comparisons test. A two-sided *P* value <0.05 was considered statistically significant.

## Supplementary Material

Supplemental Material

## Data and materials availability

All data are available in the main text or the supplementary materials.
