# Supplementary material for: Impact of vaccination on kinetics of neutralizing antibodies against SARS-CoV-2 by serum live neutralization test based on a prospective cohort
Source: Emerg Microbes Infect. 2023 Jan 19;12(1):2146535. doi: 10.1080/22221751.2022.2146535 (PMC9858416; doi:10.1080/22221751.2022.2146535)
Supplement: Supplemental Material [file TEMI_A_2146535_SM0891.docx]

**A**


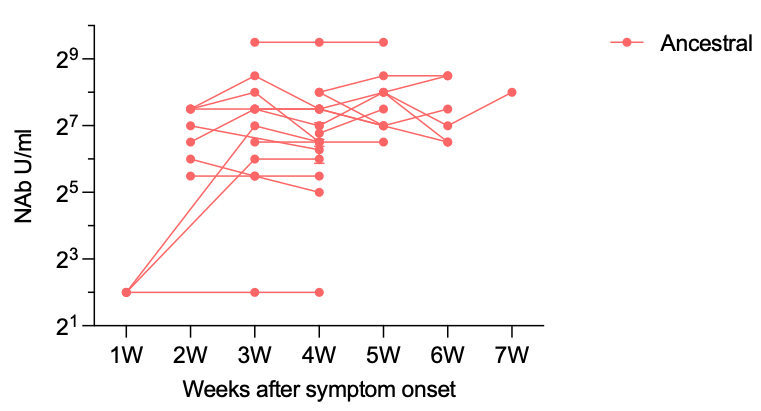


**B**


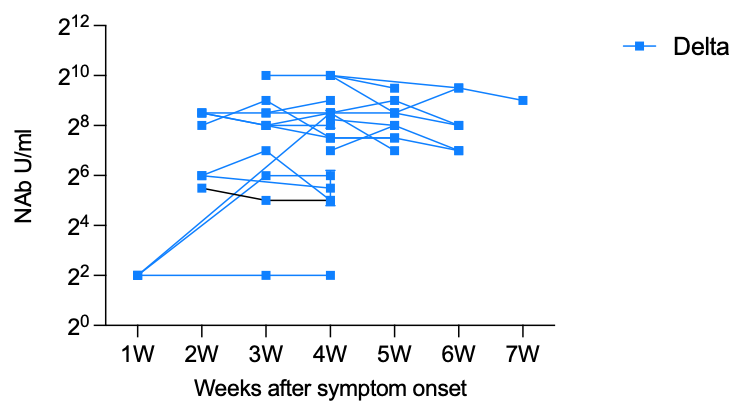


**C**


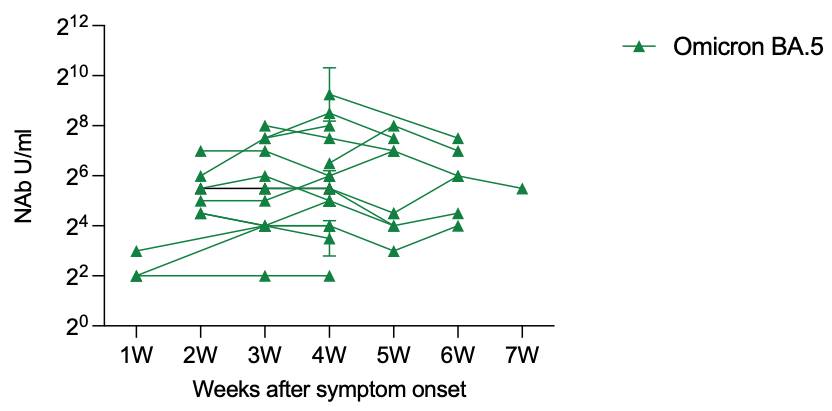


**D**


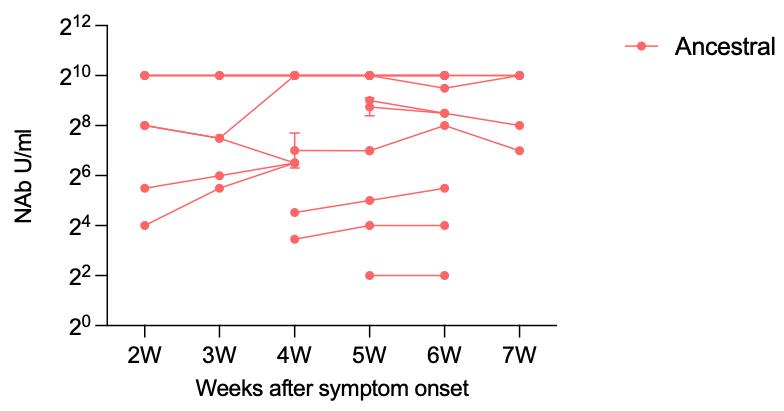


**E**


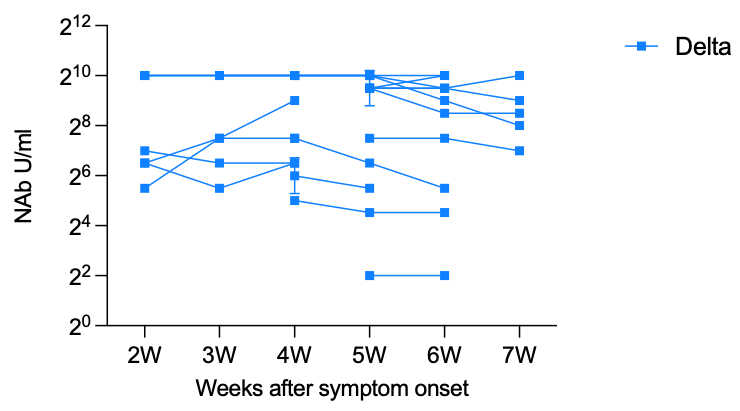


**F**


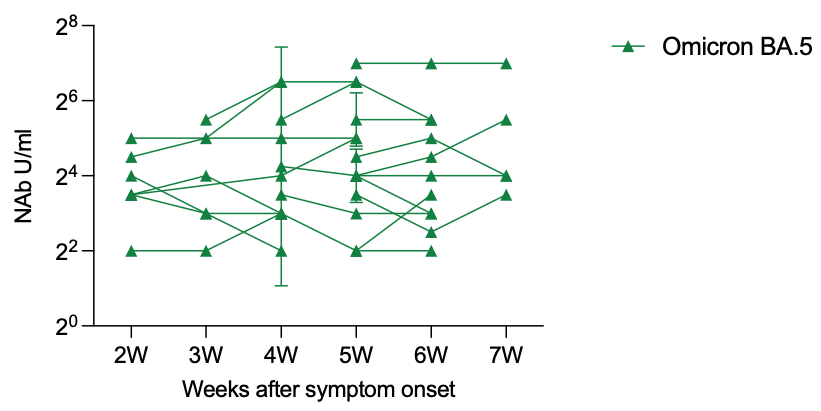


**Fig S1. Kinetics of NAb in breakthrough cases and unvaccinated-infected cases for each individual. (A)** NAbs of the ancestral strain from 1-7 weeks of the onset in breakthrough cases. **(B)** NAbs of the Delta strain from 1-7 weeks of the onset in breakthrough cases. **(C)** NAbs of the Omicron BA.5 strain from 1-7 weeks of the onset in breakthrough cases. **(D)** NAbs of the ancestral strain from 1-7 weeks of the onset in unvaccinated-infected cases. **(E)** NAbs of the Delta strain from 1-7 weeks in unvaccinated-infected cases. **(F)** NAbs of the Omicron BA.5 strain from 1-7 weeks of the onset in unvaccinated-infected cases. Each circle, square and triangle represents the titer for a serum sample.

**Supplementary data Table 1 | Sample and sequencing information.**

| **patient_id** | **sample_id** | **symptom_ onset_date** | **collection_date** | **Periods** | **symptom** | **sample_type** | **Prefecture** | **experiment_ type** | **sequencing_platform** | **Ct-ORF** | **Ct-N** | **mapped_reads** | **coverage** | **depth** |
| --- | --- | --- | --- | --- | --- | --- | --- | --- | --- | --- | --- | --- | --- | --- |
| NJ001 | NJ001 | 2021/7/19 | 2021/7/21 | NE | Mild | OPS^a^ | NJ^c^ | multiplex-pcr | BGI | 26.9 | 26.1 | 5,893,109 | 100.00% | 19715.99 |
| NJ002 | NJ002 | 2021/7/16 | 2021/7/21 | NE | Mild | OPS | NJ | multiplex-pcr | BGI | 20.55 | 19.24 | 5,891,515 | 100.00% | 19710.66 |
| NJ003 | NJ003 | 2021/7/20 | 2021/7/21 | NE | Moderate | OPS | NJ | multiplex-pcr | Illumina | 27.3 | 26.6 | 1,414,576 | 99.56% | 5493.016 |
| NJ004 | NJ004 | 2021/7/15 | 2021/7/21 | NE | Mild | OPS | NJ | multiplex-pcr | Illumina | 35.4 | 35.3 | 1,021,122 | 99.94% | 2583.658 |
| NJ005 | NJ005 | 2021/7/20 | 2021/7/21 | NE | Moderate | OPS | NJ | multiplex-pcr | Illumina | 33 | 33 | 741,008 | 99.02% | 1782.179 |
| NJ006 | NJ006 | 2021/7/17 | 2021/7/21 | NE | Moderate | OPS | NJ | multiplex-pcr | Illumina | 30.4 | 28.6 | 765,024 | 99.79% | 1936.053 |
| NJ007 | NJ007 | 2021/7/18 | 2021/7/21 | NE | Moderate | OPS | NJ | multiplex-pcr | Illumina | 33.3 | 32.1 | 856,100 | 99.41% | 2360.713 |
| NJ009 | NJ009 | 2021/7/20 | 2021/7/21 | NE | Moderate | OPS | NJ | multiplex-pcr | BGI | 31.4 | 32.1 | 5,395,751 | 100.00% | 18052.03 |
| NJ010 | NJ010 | 2021/7/15 | 2021/7/21 | NE | Mild | OPS | NJ | multiplex-pcr | Illumina | 21 | 20 | 620,017 | 99.94% | 1597.72 |
| NJ011 | NJ011 | 2021/7/18 | 2021/7/21 | NE | Moderate | OPS | NJ | multiplex-pcr | Illumina | 14 | 12 | 566,054 | 99.94% | 1478.998 |
| NJ012 | NJ012 | 2021/7/18 | 2021/7/21 | NE | Moderate | OPS | NJ | multiplex-pcr | Illumina | 18 | 19 | 1,040,647 | 99.77% | 4054.291 |
| NJ013 | NJ013 | 2021/7/20 | 2021/7/21 | NE | Moderate | OPS | NJ | multiplex-pcr | BGI | 31.6 | 28.3 | 1,906,595 | 100.00% | 6359.526 |
| NJ014 | NJ014 | 2021/7/19 | 2021/7/21 | NE | Mild | OPS | NJ | multiplex-pcr | BGI | 32 | 34 | 5,908,269 | 100.00% | 19766.71 |
| NJ015 | NJ015 | 2021/7/20 | 2021/7/21 | NE | Moderate | OPS | NJ | multiplex-pcr | Illumina | 18 | 16 | 458,247 | 99.91% | 1205.087 |
| NJ020 | NJ020 | 2021/7/13 | 2021/7/23 | NE | Mild | OPS | NJ | multiplex-pcr | Illumina | 14 | 16 | 1,491,423 | 99.94% | 4594.292 |
| NJ021 | NJ021 | 2021/7/20 | 2021/7/23 | NE | Mild | OPS | NJ | multiplex-pcr | Illumina | 26 | 26 | 1,467,781 | 99.84% | 4613.42 |
| NJ022 | NJ022 | 2021/7/20 | 2021/7/23 | NE | Moderate | OPS | NJ | multiplex-pcr | Illumina | 20 | 22 | 1,558,870 | 99.93% | 4787.205 |
| NJ023 | NJ023 | 2021/7/21 | 2021/7/23 | NE | Moderate | OPS | NJ | multiplex-pcr | Illumina | 31 | 31 | 1,070,088 | 99.43% | 3439.416 |
| NJ026 | NJ026 | 2021/7/20 | 2021/7/23 | NE | Moderate | OPS | NJ | multiplex-pcr | BGI | 30 | 30 | 2,991,657 | 99.90% | 9992.143 |
| NJ027 | NJ027 | 2021/7/20 | 2021/7/23 | NE | Moderate | OPS | NJ | multiplex-pcr | Illumina | 24 | 24 | 1,903,657 | 99.84% | 5739.518 |
| NJ028 | NJ028 | 2021/7/20 | 2021/7/23 | NE | Moderate | OPS | NJ | multiplex-pcr | BGI | 33 | 33 | 1,199,864 | 99.87% | 2009.471 |
| NJ031 | NJ031 | 2021/7/16 | 2021/7/21 | NE | Moderate | OPS | NJ | multiplex-pcr | Illumina | 33 | 30 | 2,167,484 | 99.79% | 6598.661 |
| NJ032 | NJ032 | 2021/7/13 | 2021/7/21 | NE | Moderate | OPS | NJ | multiplex-pcr | Illumina | 29 | 26 | 2,073,507 | 99.65% | 6357.679 |
| NJ033 | NJ033 | 2021/7/19 | 2021/7/23 | NE | Mild | OPS | NJ | multiplex-pcr | Illumina | 22 | 22 | 1,348,934 | 99.93% | 4229.287 |
| NJ034 | NJ034 | 2021/7/19 | 2021/7/23 | NE | Moderate | OPS | NJ | multiplex-pcr | BGI | 28 | 28 | 2,622,422 | 99.88% | 4391.188 |
| NJ035 | NJ035 | 2021/7/15 | 2021/7/23 | NE | Moderate | OPS | NJ | multiplex-pcr | BGI | 30 | 31 | 1,929,804 | 99.90% | 3230.667 |
| NJ036 | NJ036 | 2021/7/19 | 2021/7/23 | NE | Moderate | OPS | NJ | multiplex-pcr | Illumina | 19 | 20 | 1,108,812 | 99.89% | 3555.446 |
| NJ037 | NJ037 | 2021/7/17 | 2021/7/23 | NE | Moderate | OPS | NJ | multiplex-pcr | Illumina | 28 | 30 | 1,279,287 | 99.72% | 4013.909 |
| NJ039 | NJ039 | 2021/7/22 | 2021/7/23 | NE | Moderate | OPS | NJ | multiplex-pcr | Illumina | 27 | 28 | 1,313,571 | 99.83% | 4214.173 |
| NJ040 | NJ040 | 2021/7/19 | 2021/7/23 | NE | Moderate | OPS | NJ | multiplex-pcr | Illumina | 18 | 18 | 1,107,193 | 99.94% | 3582.698 |
| NJ041 | NJ041 | 2021/7/21 | 2021/7/23 | NE | Moderate | OPS | NJ | multiplex-pcr | Illumina | 25 | 25 | 1,347,369 | 99.92% | 4295.82 |
| NJ042 | NJ042 | 2021/7/21 | 2021/7/23 | NE | Mild | OPS | NJ | multiplex-pcr | Illumina | 34 | 34 | 21,798,793 | 99.94% | 67072.8 |
| NJ043 | NJ043 | 2021/7/21 | 2021/7/23 | NE | Moderate | OPS | NJ | multiplex-pcr | Illumina | 34 | 34 | 4,516,308 | 99.70% | 13911.44 |
| NJ044 | NJ044 | 2021/7/21 | 2021/7/23 | NE | Moderate | OPS | NJ | multiplex-pcr | Illumina | 17 | 18 | 3,198,293 | 99.87% | 9750.579 |
| NJ045 | NJ045 | 2021/7/21 | 2021/7/23 | NE | Moderate | OPS | NJ | multiplex-pcr | BGI | 18 | 21 | 3,572,903 | 99.90% | 11934.04 |
| NJ046 | NJ046 | 2021/7/22 | 2021/7/23 | NE | Moderate | OPS | NJ | multiplex-pcr | Illumina | 29 | 29 | 1,491,545 | 99.75% | 4432.82 |
| NJ047 | NJ047 | 2021/7/21 | 2021/7/23 | NE | Moderate | OPS | NJ | multiplex-pcr | Illumina | 25 | 25 | 1,133,726 | 99.70% | 3595.7 |
| NJ048 | NJ048 | 2021/7/21 | 2021/7/23 | NE | Moderate | OPS | NJ | multiplex-pcr | Illumina | 27 | 28 | 2,209,793 | 99.89% | 6822.537 |
| NJ049 | NJ049 | 2021/7/16 | 2021/7/23 | NE | Moderate | OPS | NJ | multiplex-pcr | Illumina | 22 | 23 | 840,602 | 99.84% | 2390.311 |
| NJ050 | NJ050 | 2021/7/19 | 2021/7/23 | NE | Moderate | OPS | NJ | multiplex-pcr | Illumina | 22 | 24 | 1,007,111 | 99.74% | 2937.674 |
| NJ051 | NJ051 | 2021/7/21 | 2021/7/23 | NE | Moderate | OPS | NJ | multiplex-pcr | Illumina | 22 | 24 | 1,077,943 | 99.77% | 3129.545 |
| NJ052 | NJ052 | 2021/7/19 | 2021/7/24 | NE | Moderate | OPS | NJ | multiplex-pcr | Illumina | 20 | 21 | 824,837 | 99.93% | 2389.559 |
| NJ053 | NJ053 | 2021/7/21 | 2021/7/24 | II | Moderate | OPS | NJ | multiplex-pcr | Illumina | 22 | 22 | 1,184,305 | 99.94% | 3440.324 |
| NJ054 | NJ054 | 2021/7/23 | 2021/7/25 | II | Moderate | OPS | NJ | multiplex-pcr | Illumina | 28 | 28 | 56,997,748 | 99.91% | 172081.2 |
| NJ055 | NJ055 | 2021/7/23 | 2021/7/25 | II | Moderate | OPS | NJ | multiplex-pcr | Illumina | 26 | 26 | 62,093,487 | 99.83% | 188204 |
| NJ056 | NJ056 | 2021/7/22 | 2021/7/25 | II | Moderate | OPS | NJ | multiplex-pcr | Illumina | 25 | 25 | 72,371,458 | 99.91% | 218513.8 |
| NJ058 | NJ058 | 2021/7/23 | 2021/7/25 | II | Moderate | OPS | NJ | multiplex-pcr | Illumina | 26 | 25 | 50,849,240 | 99.91% | 154657.7 |
| NJ059 | NJ059 | 2021/7/18 | 2021/7/25 | NE | Moderate | OPS | NJ | multiplex-pcr | Illumina | 26 | 26 | 46,140,680 | 99.94% | 140927.6 |
| NJ061 | NJ061 | 2021/7/22 | 2021/7/25 | II | Moderate | OPS | NJ | multiplex-pcr | Illumina | 28 | 28 | 64,503,228 | 99.93% | 194482.5 |
| NJ062 | NJ062 | 2021/7/23 | 2021/7/25 | II | Moderate | OPS | NJ | multiplex-pcr | Illumina | 14 | 14 | 49,430,285 | 99.94% | 151133.8 |
| NJ063 | NJ063 | 2021/7/24 | 2021/7/25 | II | Moderate | OPS | NJ | multiplex-pcr | Illumina | 19 | 19 | 76,804,846 | 99.94% | 232420.8 |
| NJ064 | NJ064 | 2021/7/24 | 2021/7/25 | II | Moderate | OPS | NJ | multiplex-pcr | Illumina | 22 | 22 | 59,360,876 | 99.94% | 180672.5 |
| NJ065 | NJ065 | 2021/7/23 | 2021/7/25 | II | Mild | OPS | NJ | multiplex-pcr | Illumina | 26 | 26 | 64,107,364 | 99.94% | 194675.3 |
| NJ066 | NJ066 | 2021/7/23 | 2021/7/25 | II | Moderate | OPS | NJ | multiplex-pcr | Illumina | 16 | 16 | 43,046,948 | 99.94% | 132379.2 |
| NJ067 | NJ067 | 2021/7/22 | 2021/7/25 | II | Moderate | OPS | NJ | multiplex-pcr | Illumina | 20 | 21 | 60,190,823 | 99.94% | 183448.2 |
| NJ068 | NJ068 | 2021/7/22 | 2021/7/25 | II | Moderate | OPS | NJ | multiplex-pcr | Illumina | 20 | 22 | 46,339,293 | 99.94% | 140256.7 |
| NJ070 | NJ070 | 2021/7/22 | 2021/7/25 | II | Moderate | OPS | NJ | multiplex-pcr | Illumina | 18 | 20 | 71,347,296 | 99.94% | 216842.6 |
| NJ071 | NJ071 | 2021/7/24 | 2021/7/25 | II | Moderate | OPS | NJ | multiplex-pcr | Illumina | 17 | 17 | 47,620,887 | 99.94% | 145410.3 |
| NJ073 | NJ073 | 2021/7/22 | 2021/7/25 | II | Moderate | OPS | NJ | multiplex-pcr | Illumina | 33 | 33 | 1,716,750 | 99.94% | 5737.751 |
| NJ074 | NJ074 | 2021/7/22 | 2021/8/8 | NE | Moderate | OPS | NJ | multiplex-pcr | Illumina | 30 | 30 | 435,812 | 99.65% | 1766.879 |
| NJ075 | NJ075 | 2021/7/21 | 2021/7/31 | II | Moderate | OPS | NJ | multiplex-pcr | Illumina | 24 | 26 | 15,848,415 | 99.91% | 58279.28 |
| NJ078 | NJ078 | 2021/7/23 | 2021/7/31 | II | Moderate | OPS | NJ | multiplex-pcr | Illumina | 32 | 34 | 8,925,398 | 99.88% | 31245.42 |
| NJ079 | NJ079 | 2021/7/25 | 2021/7/31 | II | Mild | OPS | NJ | multiplex-pcr | Illumina | 34 | 36 | 10,381,604 | 99.92% | 32105.38 |
| NJ080 | NJ080 | 2021/7/25 | 2021/7/31 | II | Moderate | OPS | NJ | multiplex-pcr | Illumina | 22 | 24 | 26,219,270 | 99.94% | 100515 |
| NJ082 | NJ082 | 2021/7/22 | 2021/7/31 | II | Mild | OPS | NJ | multiplex-pcr | Illumina | 17 | 23 | 16,428,932 | 99.94% | 64312.04 |
| NJ084 | NJ084 | 2021/7/23 | 2021/7/31 | II | Moderate | OPS | NJ | multiplex-pcr | Illumina | 17 | 23 | 28,543,584 | 99.94% | 111964.9 |
| NJ085 | NJ085 | 2021/7/21 | 2021/7/31 | II | Moderate | OPS | NJ | multiplex-pcr | Illumina | 17 | 22 | 18,836,610 | 99.94% | 73941.13 |
| NJ086 | NJ086 | 2021/7/25 | 2021/7/31 | II | Moderate | OPS | NJ | multiplex-pcr | Illumina | 23 | 25 | 32,857,352 | 99.94% | 127995.6 |
| NJ087 | NJ087 | 2021/7/22 | 2021/7/31 | II | Mild | OPS | NJ | multiplex-pcr | Illumina | 19 | 21 | 49,255,674 | 99.94% | 190979.2 |
| NJ088 | NJ088 | 2021/7/25 | 2021/7/31 | II | Moderate | OPS | NJ | multiplex-pcr | Illumina | 15 | 17 | 38,257,880 | 99.94% | 150158.5 |
| NJ089 | NJ089 | 2021/7/23 | 2021/7/31 | II | Mild | OPS | NJ | multiplex-pcr | Illumina | 32 | 23 | 5,358,593 | 99.88% | 19284.6 |
| NJ090 | NJ090 | 2021/7/24 | 2021/7/31 | II | Moderate | OPS | NJ | multiplex-pcr | Illumina | 21 | 25 | 29,846,203 | 99.94% | 116494.4 |
| NJ091 | NJ091 | 2021/7/25 | 2021/7/31 | II | Moderate | OPS | NJ | multiplex-pcr | Illumina | 21 | 24 | 9,941,793 | 99.94% | 39078.62 |
| NJ092 | NJ092 | 2021/7/24 | 2021/7/31 | II | Mild | OPS | NJ | multiplex-pcr | Illumina | 24 | 27 | 12,788,936 | 99.94% | 49801.79 |
| NJ093 | NJ093 | 2021/7/23 | 2021/7/31 | II | Moderate | OPS | NJ | multiplex-pcr | Illumina | 22 | 24 | 15,893,058 | 99.94% | 57446.12 |
| NJ094 | NJ094 | 2021/7/25 | 2021/7/31 | II | Moderate | OPS | NJ | multiplex-pcr | Illumina | 22 | 22 | 8,984,801 | 99.94% | 34554.35 |
| NJ095 | NJ095 | 2021/7/25 | 2021/7/31 | II | Mild | OPS | NJ | multiplex-pcr | Illumina | 16 | 18 | 15,173,624 | 99.94% | 58000.42 |
| NJ096 | NJ096 | 2021/7/25 | 2021/7/31 | II | Moderate | OPS | NJ | multiplex-pcr | Illumina | 14 | 17 | 9,733,624 | 99.94% | 36982.72 |
| NJ097 | NJ097 | 2021/7/24 | 2021/7/31 | II | Moderate | OPS | NJ | multiplex-pcr | Illumina | 23 | 24 | 11,363,700 | 99.94% | 42677.63 |
| NJ098 | NJ098 | 2021/7/25 | 2021/7/31 | II | Moderate | OPS | NJ | multiplex-pcr | Illumina | 32 | 34 | 12,737,216 | 99.93% | 46090.27 |
| NJ099 | NJ099 | 2021/7/25 | 2021/7/31 | II | Mild | OPS | NJ | multiplex-pcr | Illumina | 18 | 21 | 10,816,901 | 99.91% | 41025.49 |
| NJ100 | NJ100 | 2021/7/24 | 2021/7/31 | II | Moderate | OPS | NJ | multiplex-pcr | Illumina | 28 | 29 | 23,993,151 | 99.88% | 92676.05 |
| NJ101 | NJ101 | 2021/7/25 | 2021/7/31 | II | Moderate | OPS | NJ | multiplex-pcr | Illumina | 15 | 18 | 9,730,420 | 99.94% | 36687.12 |
| NJ102 | NJ102 | 2021/7/25 | 2021/7/31 | II | Mild | OPS | NJ | multiplex-pcr | Illumina | 17 | 17 | 12,225,834 | 99.94% | 47861.34 |
| NJ103 | NJ103 | 2021/7/19 | 2021/7/26 | NE | Moderate | OPS | NJ | multiplex-pcr | BGI | 22 | 22 | 2,618,222 | 99.94% | 5256.675 |
| NJ104 | NJ104 | 2021/7/25 | 2021/7/31 | II | Moderate | OPS | NJ | multiplex-pcr | Illumina | 19 | 21 | 8,980,819 | 99.94% | 34307.58 |
| NJ105 | NJ105 | 2021/7/25 | 2021/7/31 | II | Moderate | OPS | NJ | multiplex-pcr | Illumina | 30 | 32 | 15,530,829 | 99.85% | 53571.21 |
| NJ106 | NJ106 | 2021/7/24 | 2021/7/31 | II | Moderate | OPS | NJ | multiplex-pcr | Illumina | 16 | 16 | 14,215,135 | 99.94% | 52387.41 |
| NJ107 | NJ107 | 2021/7/25 | 2021/7/31 | II | Moderate | OPS | NJ | multiplex-pcr | Illumina | 16 | 19 | 12,906,137 | 99.94% | 47243.8 |
| NJ110 | NJ110 | 2021/7/25 | 2021/7/31 | II | Mild | OPS | NJ | multiplex-pcr | Illumina | 31 | 31 | 10,273,633 | 99.88% | 30893.7 |
| NJ114 | NJ114 | 2021/7/24 | 2021/7/27 | II | Mild | OPS | NJ | multiplex-pcr | Illumina | 20 | 19 | 4,177,539 | 99.94% | 11840.62 |
| NJ115 | NJ115 | 2021/7/26 | 2021/7/27 | II | Mild | OPS | NJ | multiplex-pcr | Illumina | 34 | 32 | 1,381,603 | 99.75% | 3748.584 |
| NJ120 | NJ120 | 2021/7/24 | 2021/7/31 | II | Moderate | OPS | NJ | multiplex-pcr | Illumina | 28.8 | 25.9 | 933,030 | 99.55% | 3555.006 |
| NJ121 | NJ121 | 2021/7/22 | 2021/7/31 | II | Moderate | OPS | NJ | multiplex-pcr | BGI | 38.7 | 35.9 | 94,759 | 99.65% | 316.3816 |
| NJ124 | NJ124 | 2021/7/22 | 2021/7/31 | II | Moderate | OPS | NJ | multiplex-pcr | Illumina | 18.2 | 15.3 | 368,032 | 99.80% | 12.33765 |
| NJ125 | NJ125 | 2021/7/23 | 2021/7/31 | II | Moderate | OPS | NJ | multiplex-pcr | Illumina | 20.4 | 17.5 | 640,625 | 99.61% | 1929.753 |
| NJ126 | NJ126 | 2021/7/26 | 2021/7/31 | II | Moderate | OPS | NJ | multiplex-pcr | Illumina | 15.7 | 13.2 | 347,775 | 99.59% | 1013.313 |
| NJ127 | NJ127 | 2021/7/24 | 2021/7/31 | II | Moderate | OPS | NJ | multiplex-pcr | Illumina | 30 | 29 | 18,652,075 | 99.94% | 67441.5 |
| NJ129 | NJ129 | 2021/7/26 | 2021/7/31 | II | Moderate | OPS | NJ | multiplex-pcr | Illumina | 23.4 | 20.7 | 1,265,743 | 99.52% | 4981.298 |
| NJ131 | NJ131 | 2021/7/25 | 2021/7/31 | II | Mild | OPS | NJ | multiplex-pcr | Illumina | 17.7 | 14.7 | 956,763 | 99.64% | 2587.101 |
| NJ132 | NJ132 | 2021/7/24 | 2021/7/31 | II | Moderate | OPS | NJ | multiplex-pcr | Illumina | 25 | 25 | 16,835,744 | 99.94% | 62428.16 |
| NJ133 | NJ133 | 2021/7/26 | 2021/7/31 | II | Mild | OPS | NJ | multiplex-pcr | Illumina | 29 | 29 | 15,810,833 | 99.94% | 57493.49 |
| NJ137 | NJ137 | 2021/7/26 | 2021/8/10 | II | Moderate | OPS | NJ | multiplex-pcr | Illumina | 26 | 26 | 2,232,338 | 99.85% | 7913.897 |
| NJ138 | NJ138 | 2021/7/26 | 2021/7/31 | II | Moderate | OPS | NJ | multiplex-pcr | Illumina | 23.1 | 22 | 264,942 | 99.54% | 1040.55 |
| NJ141 | NJ141 | 2021/7/26 | 2021/7/31 | II | Moderate | OPS | NJ | multiplex-pcr | Illumina | 18.8 | 15.6 | 980,602 | 99.52% | 2828.862 |
| NJ142 | NJ142 | 2021/7/26 | 2021/7/31 | II | Moderate | OPS | NJ | multiplex-pcr | Illumina | 31 | 31 | 5,402,126 | 99.92% | 8195.749 |
| NJ143 | NJ143 | 2021/7/26 | 2021/8/10 | II | Mild | OPS | NJ | multiplex-pcr | TF^g^ | 34 | 34 | 220,922 | 99.73% | 1464.925 |
| NJ145 | NJ145 | 2021/7/26 | 2021/7/31 | II | Mild | OPS | NJ | multiplex-pcr | Illumina | 22.9 | 19.5 | 738,808 | 99.59% | 2284.6 |
| NJ146 | NJ146 | 2021/7/25 | 2021/7/31 | II | Moderate | OPS | NJ | multiplex-pcr | Illumina | 24 | 20.5 | 494,037 | 99.61% | 1530.478 |
| NJ152 | NJ152 | 2021/7/26 | 2021/8/10 | II | Moderate | OPS | NJ | multiplex-pcr | TF | 25 | 25 | 2,030,437 | 99.80% | 13709.94 |
| NJ156 | NJ156 | 2021/7/26 | 2021/7/31 | II | Moderate | OPS | NJ | multiplex-pcr | Illumina | 24.8 | 23.4 | 950,238 | 99.64% | 2868 |
| NJ157 | NJ157 | 2021/7/27 | 2021/7/31 | CI | Moderate | OPS | NJ | multiplex-pcr | Illumina | 33.5 | 32 | 1,036,888 | 99.43% | 3196.759 |
| NJ158 | NJ158 | 2021/7/25 | 2021/7/31 | II | Moderate | OPS | NJ | multiplex-pcr | Illumina | 33.3 | 31.5 | 758,709 | 99.46% | 2321.692 |
| NJ159 | NJ159 | 2021/7/27 | 2021/7/28 | CI | Moderate | OPS | NJ | multiplex-pcr | Illumina | 12 | 13 | 7,972,661 | 99.94% | 22378.86 |
| NJ160 | NJ160 | 2021/7/26 | 2021/7/27 | II | Moderate | OPS | NJ | multiplex-pcr | BGI | 18 | 22 | 4,165,046 | 99.88% | 8368.15 |
| NJ162 | NJ162 | 2021/7/27 | 2021/7/31 | CI | Moderate | OPS | NJ | multiplex-pcr | Illumina | 21.2 | 19.4 | 930,888 | 99.73% | 2839.635 |
| NJ164 | NJ164 | 2021/7/26 | 2021/8/10 | II | Moderate | OPS | NJ | multiplex-pcr | Illumina | 26 | 27 | 206,891 | 99.45% | 811.6303 |
| NJ165 | NJ165 | 2021/7/28 | 2021/7/31 | CI | Moderate | OPS | NJ | multiplex-pcr | Illumina | 20.3 | 19.3 | 207,893 | 99.31% | 614.5542 |
| NJ166 | NJ166 | 2021/7/27 | 2021/7/31 | CI | Moderate | OPS | NJ | multiplex-pcr | BGI | 36.1 | 36.7 | 170,059 | 99.78% | 567.0518 |
| NJ167 | NJ167 | 2021/7/27 | 2021/7/31 | CI | Moderate | OPS | NJ | multiplex-pcr | Illumina | 28.6 | 29.5 | 1,994,734 | 99.56% | 7735.701 |
| NJ168 | NJ168 | 2021/7/27 | 2021/7/31 | CI | Moderate | OPS | NJ | multiplex-pcr | Illumina | 29.2 | 28 | 1,981,999 | 99.57% | 7442.95 |
| NJ175 | NJ175 | 2021/7/27 | 2021/7/31 | CI | Mild | OPS | NJ | multiplex-pcr | Illumina | 25.4 | 23.9 | 1,148,512 | 99.68% | 3378.314 |
| NJ176 | NJ176 | 2021/7/27 | 2021/7/31 | CI | Mild | OPS | NJ | multiplex-pcr | Illumina | 29.4 | 27.8 | 1,615,010 | 99.52% | 6120.011 |
| NJ177 | NJ177 | 2021/7/28 | 2021/7/31 | CI | Moderate | OPS | NJ | multiplex-pcr | Illumina | 29.1 | 28.1 | 915,298 | 99.54% | 3627.126 |
| NJ179 | NJ179 | 2021/7/28 | 2021/7/31 | CI | Moderate | OPS | NJ | multiplex-pcr | Illumina | 20.4 | 18.3 | 1,348,116 | 99.78% | 4041.57 |
| NJ182 | NJ182 | 2021/7/29 | 2021/7/31 | CI | Moderate | OPS | NJ | multiplex-pcr | Illumina | 27.2 | 24.6 | 2,025,271 | 99.56% | 7723.182 |
| NJ184 | NJ184 | 2021/7/28 | 2021/7/31 | CI | Moderate | OPS | NJ | multiplex-pcr | BGI | 36.4 | 35.2 | 327,835 | 99.89% | 1093.516 |
| NJ186 | NJ186 | 2021/7/29 | 2021/8/10 | CI | Moderate | OPS | NJ | multiplex-pcr | Illumina | 33 | 33 | 24,917 | 98.92% | 100.0977 |
| NJ187 | NJ187 | 2021/7/29 | 2021/7/31 | CI | Moderate | OPS | NJ | multiplex-pcr | Illumina | 21.8 | 20.8 | 1,040,421 | 99.67% | 3154.95 |
| NJ188 | NJ188 | 2021/7/28 | 2021/7/31 | CI | Moderate | OPS | NJ | multiplex-pcr | Illumina | 27.9 | 26.5 | 1,326,499 | 99.55% | 5174.659 |
| NJ189 | NJ189 | 2021/7/30 | 2021/7/31 | CI | Mild | OPS | NJ | multiplex-pcr | Illumina | 27.9 | 26.4 | 1,061,889 | 99.62% | 3186.831 |
| NJ192 | NJ192 | 2021/7/30 | 2021/7/31 | CI | Moderate | OPS | NJ | multiplex-pcr | Illumina | 25.4 | 23.9 | 1,508,078 | 99.60% | 5824.268 |
| NJ193 | NJ193 | 2021/7/30 | 2021/8/10 | CI | Mild | OPS | NJ | multiplex-pcr | TF | 30 | 30 | 2,329,786 | 99.75% | 15682.61 |
| NJ195 | NJ195 | 2021/7/24 | 2021/7/31 | II | Moderate | OPS | NJ | multiplex-pcr | TF | 39 | 39 | 2,139,455 | 99.86% | 14813.98 |
| NJ196 | NJ196 | 2021/7/28 | 2021/8/3 | CI | Moderate | OPS | NJ | multiplex-pcr | TF | 32 | 30 | 2,686,673 | 99.88% | 15400.34 |
| NJ197 | NJ197 | 2021/7/30 | 2021/8/3 | CI | Mild | OPS | NJ | multiplex-pcr | TF | 25 | 27 | 3,707,905 | 99.89% | 25547.63 |
| NJ198 | NJ198 | 2021/7/30 | 2021/8/3 | CI | Moderate | OPS | NJ | multiplex-pcr | TF | 26 | 27 | 647,062 | 99.82% | 4427.391 |
| NJ199 | NJ199 | 2021/7/31 | 2021/8/10 | CI | Moderate | OPS | NJ | multiplex-pcr | TF | 30 | 30 | 1,365,684 | 99.78% | 9089.513 |
| NJ200 | NJ200 | 2021/7/31 | 2021/8/3 | CI | Moderate | OPS | NJ | multiplex-pcr | TF | 22 | 24 | 5,636,384 | 99.83% | 39085.66 |
| NJ201 | NJ201 | 2021/7/31 | 2021/8/3 | CI | Moderate | OPS | NJ | multiplex-pcr | TF | 25 | 27 | 2,350,858 | 99.83% | 16234.55 |
| NJ203 | NJ203 | 2021/7/30 | 2021/8/10 | CI | Moderate | OPS | NJ | multiplex-pcr | TF | 33 | 33 | 1,372,493 | 99.78% | 9263.599 |
| NJ204 | NJ204 | 2021/7/28 | 2021/8/3 | CI | Mild | OPS | NJ | multiplex-pcr | TF | 23 | 25 | 4,055,842 | 99.85% | 28033.93 |
| NJ205 | NJ205 | 2021/7/31 | 2021/8/3 | CI | Mild | OPS | NJ | multiplex-pcr | TF | 26 | 28 | 1,019,838 | 99.79% | 7040.493 |
| NJ206 | NJ206 | 2021/7/31 | 2021/8/3 | CI | Moderate | OPS | NJ | multiplex-pcr | TF | 18 | 20 | 5,428,442 | 99.92% | 29739.42 |
| NJ207 | NJ207 | 2021/7/31 | 2021/8/3 | CI | Moderate | OPS | NJ | multiplex-pcr | TF | 14 | 16 | 5,778,873 | 99.83% | 37018.06 |
| NJ208 | NJ208 | 2021/7/22 | 2021/8/3 | II | Mild | OPS | NJ | multiplex-pcr | TF | 32 | 33 | 1,654,095 | 99.82% | 10618.64 |
| NJ209 | NJ209 | 2021/7/30 | 2021/8/3 | CI | Moderate | OPS | NJ | multiplex-pcr | TF | 24 | 27 | 4,016,786 | 99.84% | 25074.3 |
| NJ210 | NJ210 | 2021/7/30 | 2021/8/3 | CI | Mild | OPS | NJ | multiplex-pcr | TF | 17 | 20 | 3,074,352 | 99.78% | 17238.55 |
| NJ211 | NJ211 | 2021/7/29 | 2021/8/3 | CI | Moderate | OPS | NJ | multiplex-pcr | TF | 23 | 24 | 3,905,947 | 99.82% | 26785.25 |
| NJ212 | NJ212 | 2021/7/31 | 2021/8/3 | CI | Mild | OPS | NJ | multiplex-pcr | TF | 17 | 15 | 3,038,581 | 99.83% | 15513.77 |
| NJ213 | NJ213 | 2021/7/31 | 2021/8/3 | CI | Mild | OPS | NJ | multiplex-pcr | TF | 37 | 36 | 1,195,453 | 99.56% | 5174.975 |
| NJ215 | NJ215 | 2021/7/31 | 2021/8/3 | CI | Moderate | OPS | NJ | multiplex-pcr | TF | 31 | 33 | 2,874,080 | 99.87% | 18781.26 |
| NJ217 | NJ217 | 2021/7/31 | 2021/8/3 | CI | Moderate | OPS | NJ | multiplex-pcr | TF | 13 | 15 | 4,053,568 | 99.92% | 24870.71 |
| NJ218 | NJ218 | 2021/8/1 | 2021/8/10 | CI | Moderate | OPS | NJ | multiplex-pcr | TF | 28 | 29 | 2,451,953 | 99.79% | 16057.24 |
| NJ220 | NJ220 | 2021/7/31 | 2021/8/3 | CI | Moderate | OPS | NJ | multiplex-pcr | TF | 15 | 19 | 4,713,137 | 99.84% | 30434.29 |
| NJ221 | NJ221 | 2021/8/2 | 2021/8/10 | CI | Moderate | OPS | NJ | multiplex-pcr | TF | 30 | 30 | 1,979,837 | 99.78% | 13042.76 |
| NJ222 | NJ222 | 2021/7/30 | 2021/8/6 | CI | Moderate | OPS | NJ | multiplex-pcr | TF | 18 | 20 | 2,912,071 | 99.67% | 16475.41 |
| NJ223 | NJ223 | 2021/7/26 | 2021/8/6 | CI | Mild | NPS^b^ | NJ | multiplex-pcr | Illumina | 30 | 30 | 335,666 | 98.79% | 1037.277 |
| NJ224 | NJ224 | 2021/8/2 | 2021/8/6 | CI | Moderate | NPS | NJ | multiplex-pcr | Illumina | 16 | 19 | 736,787 | 99.59% | 2887.592 |
| NJ226 | NJ226 | 2021/8/1 | 2021/8/4 | CI | Moderate | OPS | NJ | multiplex-pcr | Illumina | 35 | 33 | 2,021,475 | 99.76% | 7442.81 |
| NJ227 | NJ227 | 2021/8/3 | 2021/8/4 | CI | Moderate | OPS | NJ | multiplex-pcr | Illumina | 34 | 33 | 1,193,633 | 99.70% | 4344.945 |
| NJ228 | NJ228 | 2021/8/4 | 2021/8/6 | CI | Moderate | OPS | NJ | multiplex-pcr | Illumina | 38 | 36 | 862,254 | 99.87% | 3436.348 |
| NJ229 | NJ229 | 2021/8/5 | 2021/8/6 | ER | Moderate | NPS | NJ | multiplex-pcr | Illumina | 36 | 36 | 670,441 | 99.41% | 2329.684 |
| NJ230 | NJ230 | 2021/8/6 | 2021/8/7 | ER | Moderate | NPS | NJ | multiplex-pcr | Illumina | 19 | 18 | 544,726 | 99.82% | 1882.677 |
| NJ232 | NJ232 | 2021/8/8 | 2021/8/9 | ER | Moderate | OPS | NJ | multiplex-pcr | Illumina | 24 | 25 | 23,233,671 | 99.93% | 90935.46 |
| NJ234 | NJ234 | 2021/8/10 | 2021/8/14 | ER | Moderate | OPS | NJ | multiplex-pcr | Illumina | 20 | 21 | 1,643,699 | 99.94% | 6532.425 |
| NJ235 | NJ235 | 2021/8/11 | 2021/8/13 | ER | Moderate | OPS | NJ | multiplex-pcr | BGI | 28 | 26 | 12,826,523 | 99.93% | 40712.12 |
| YZ001 | YZ001 | 2021/7/23 | 2021/7/28 | NE | Moderate | OPS | YZ^d^ | multiplex-pcr | Illumina | 19 | 20 | 2,712,182 | 99.92% | 7319.275 |
| YZ002 | YZ002 | 2021/7/25 | 2021/8/2 | NE | Moderate | OPS | YZ | multiplex-pcr | Illumina | 16 | 16 | 58,468,029 | 99.93% | 165219.4 |
| YZ003 | YZ003 | 2021/7/25 | 2021/8/2 | NE | Moderate | OPS | YZ | multiplex-pcr | Illumina | 18 | 18 | 36,241,018 | 99.92% | 100101.2 |
| YZ004 | YZ004 | 2021/7/27 | 2021/8/2 | NE | Moderate | OPS | YZ | multiplex-pcr | Illumina | 18 | 18 | 8,596,632 | 99.92% | 17094.83 |
| YZ005 | YZ005 | 2021/7/28 | 2021/8/2 | NE | Moderate | OPS | YZ | multiplex-pcr | Illumina | 18 | 18 | 32,763,885 | 99.94% | 90837.97 |
| YZ006 | YZ006 | 2021/7/25 | 2021/8/2 | NE | Moderate | OPS | YZ | multiplex-pcr | BGI | 18 | 19 | 9,629,323 | 99.87% | 23400.49 |
| YZ007 | YZ007 | 2021/7/28 | 2021/8/2 | NE | Moderate | OPS | YZ | multiplex-pcr | Illumina | 30 | 30 | 52,444,838 | 99.93% | 137320 |
| YZ009 | YZ009 | 2021/7/25 | 2021/8/2 | NE | Moderate | OPS | YZ | multiplex-pcr | Illumina | 18 | 14 | 69,834,847 | 99.92% | 177166.5 |
| YZ010 | YZ010 | 2021/7/27 | 2021/8/10 | NE | Severe | OPS | YZ | multiplex-pcr | Illumina | 26 | 26 | 1,347,834 | 99.30% | 5408.447 |
| YZ012 | YZ012 | 2021/7/26 | 2021/8/2 | NE | Moderate | OPS | YZ | multiplex-pcr | Illumina | 20 | 18 | 44,594,971 | 99.92% | 113449 |
| YZ013 | YZ013 | 2021/7/29 | 2021/8/2 | NE | Moderate | OPS | YZ | multiplex-pcr | Illumina | 18 | 18 | 57,395,337 | 99.92% | 153102.3 |
| YZ014 | YZ014 | 2021/7/29 | 2021/8/2 | NE | Critical | OPS | YZ | multiplex-pcr | Illumina | 36 | 34 | 64,707,878 | 99.92% | 176122.7 |
| YZ015 | YZ015 | 2021/7/28 | 2021/8/2 | NE | Critical | OPS | YZ | multiplex-pcr | Illumina | 28 | 28 | 26,392,447 | 99.92% | 54900.49 |
| YZ016 | YZ016 | 2021/7/28 | 2021/8/2 | NE | Moderate | OPS | YZ | multiplex-pcr | Illumina | 17 | 17 | 2,917,680 | 99.94% | 10812.31 |
| YZ018 | YZ018 | 2021/7/29 | 2021/8/2 | NE | Moderate | NPS | YZ | multiplex-pcr | Illumina | 16 | 16 | 15,010,240 | 99.87% | 55382.95 |
| YZ019 | YZ019 | 2021/7/29 | 2021/8/4 | NE | Severe | OPS | YZ | multiplex-pcr | TF | 30 | 31 | 1,779,230 | 99.75% | 12514.63 |
| YZ020 | YZ020 | 2021/7/28 | 2021/8/2 | NE | Moderate | OPS | YZ | multiplex-pcr | Illumina | 18 | 18 | 2,227,180 | 99.94% | 8663.303 |
| YZ021 | YZ021 | 2021/7/24 | 2021/8/2 | NE | Moderate | OPS | YZ | multiplex-pcr | Illumina | 30 | 30 | 16,018,498 | 99.94% | 62475.28 |
| YZ023 | YZ023 | 2021/7/26 | 2021/8/2 | NE | Moderate | OPS | YZ | multiplex-pcr | Illumina | 14 | 12 | 13,963,501 | 99.94% | 54454.75 |
| YZ025 | YZ025 | 2021/7/29 | 2021/8/2 | NE | Mild | OPS | YZ | multiplex-pcr | Illumina | 26 | 26 | 2,812,766 | 99.93% | 10640.39 |
| YZ026 | YZ026 | 2021/7/24 | 2021/8/2 | NE | Moderate | OPS | YZ | multiplex-pcr | Illumina | 28 | 26 | 2,584,161 | 99.94% | 9893.152 |
| YZ029 | YZ029 | 2021/7/29 | 2021/8/2 | NE | Moderate | OPS | YZ | multiplex-pcr | Illumina | 16 | 16 | 21,371,244 | 99.94% | 82914.15 |
| YZ030 | YZ030 | 2021/7/30 | 2021/8/2 | II | Moderate | OPS | YZ | multiplex-pcr | BGI | 30 | 30 | 611,093 | 99.77% | 1479.57 |
| YZ031 | YZ031 | 2021/7/30 | 2021/8/2 | II | Critical | OPS | YZ | multiplex-pcr | BGI | 28 | 27 | 7,674,117 | 99.87% | 18306.22 |
| YZ032 | YZ032 | 2021/7/31 | 2021/8/2 | II | Moderate | OPS | YZ | multiplex-pcr | BGI | 30 | 28 | 1,875,242 | 99.82% | 4569.217 |
| YZ033 | YZ033 | 2021/7/30 | 2021/8/4 | NE | Moderate | OPS | YZ | multiplex-pcr | TF | 30 | 29 | 3,253,177 | 99.81% | 19820.28 |
| YZ034 | YZ034 | 2021/7/30 | 2021/8/2 | NE | Moderate | OPS | YZ | multiplex-pcr | BGI | 24 | 25 | 9,561,798 | 99.87% | 23011.31 |
| YZ035 | YZ035 | 2021/7/28 | 2021/8/2 | NE | Critical | OPS | YZ | multiplex-pcr | BGI | 33 | 33 | 4,420,638 | 99.76% | 10363.18 |
| YZ036 | YZ036 | 2021/7/29 | 2021/8/2 | NE | Moderate | OPS | YZ | multiplex-pcr | BGI | 11 | 13 | 3,511,111 | 99.87% | 8343.174 |
| YZ037 | YZ037 | 2021/7/30 | 2021/8/2 | II | Moderate | OPS | YZ | multiplex-pcr | Illumina | 24 | 24 | 21,821,627 | 99.94% | 85312.91 |
| YZ038 | YZ038 | 2021/7/28 | 2021/8/2 | NE | Moderate | OPS | YZ | multiplex-pcr | Illumina | 20 | 20 | 2,683,209 | 99.94% | 10163.03 |
| YZ039 | YZ039 | 2021/7/31 | 2021/8/2 | II | Moderate | OPS | YZ | multiplex-pcr | Illumina | 36 | 32 | 17,285,078 | 99.94% | 66818.34 |
| YZ041 | YZ041 | 2021/7/30 | 2021/8/2 | NE | Moderate | OPS | YZ | multiplex-pcr | Illumina | 16 | 14 | 9,218,644 | 99.90% | 35912.87 |
| YZ043 | YZ043 | 2021/7/29 | 2021/8/2 | NE | Moderate | OPS | YZ | multiplex-pcr | Illumina | 20 | 20 | 10,396,251 | 99.93% | 39480.57 |
| YZ044 | YZ044 | 2021/7/28 | 2021/8/2 | NE | Moderate | OPS | YZ | multiplex-pcr | Illumina | 22 | 22 | 15,465,439 | 99.94% | 60372.59 |
| YZ045 | YZ045 | 2021/7/28 | 2021/8/2 | NE | Critical | OPS | YZ | multiplex-pcr | Illumina | 22 | 22 | 11,045,899 | 99.93% | 42928.45 |
| YZ046 | YZ046 | 2021/7/29 | 2021/8/2 | NE | Moderate | OPS | YZ | multiplex-pcr | Illumina | 26 | 26 | 8,661,265 | 99.94% | 34165.79 |
| YZ047 | YZ047 | 2021/7/30 | 2021/8/2 | II | Moderate | OPS | YZ | multiplex-pcr | Illumina | 26 | 26 | 8,709,930 | 99.94% | 33456.66 |
| YZ048 | YZ048 | 2021/7/29 | 2021/8/2 | NE | Mild | OPS | YZ | multiplex-pcr | BGI | 35 | 34 | 128,185 | 99.75% | 305.8903 |
| YZ049 | YZ049 | 2021/7/26 | 2021/8/2 | NE | Moderate | OPS | YZ | multiplex-pcr | Illumina | 18 | 16 | 9,749,776 | 99.94% | 37718.75 |
| YZ050 | YZ050 | 2021/7/28 | 2021/8/2 | NE | Moderate | OPS | YZ | multiplex-pcr | Illumina | 30 | 28 | 10,313,725 | 99.94% | 40392.38 |
| YZ051 | YZ051 | 2021/7/25 | 2021/8/2 | NE | Moderate | OPS | YZ | multiplex-pcr | BGI | 15 | 16 | 8,365,427 | 99.87% | 19913.8 |
| YZ052 | YZ052 | 2021/7/30 | 2021/8/2 | NE | Moderate | OPS | YZ | multiplex-pcr | Illumina | 30 | 24 | 15,001,500 | 99.94% | 57833.11 |
| YZ053 | YZ053 | 2021/7/29 | 2021/8/2 | NE | Moderate | OPS | YZ | multiplex-pcr | Illumina | 26 | 24 | 12,749,496 | 99.94% | 49413.07 |
| YZ054 | YZ054 | 2021/7/30 | 2021/8/2 | NE | Moderate | OPS | YZ | multiplex-pcr | Illumina | 32 | 32 | 10,703,647 | 99.94% | 42188.11 |
| YZ056 | YZ056 | 2021/7/31 | 2021/8/4 | II | Moderate | OPS | YZ | multiplex-pcr | TF | 29 | 29.6 | 1,760,781 | 99.81% | 12293.7 |
| YZ057 | YZ057 | 2021/7/31 | 2021/8/2 | II | Critical | OPS | YZ | multiplex-pcr | BGI | 33 | 33 | 2,299,706 | 99.82% | 5468.761 |
| YZ058 | YZ058 | 2021/7/31 | 2021/8/2 | II | Severe | OPS | YZ | multiplex-pcr | TF | 10 | 9 | 6,754,215 | 99.94% | 42702.32 |
| YZ059 | YZ059 | 2021/7/29 | 2021/8/2 | II | Moderate | OPS | YZ | multiplex-pcr | TF | 30.8 | 29.5 | 366,294 | 99.82% | 2137.946 |
| YZ060 | YZ060 | 2021/7/28 | 2021/8/10 | NE | Moderate | OPS | YZ | multiplex-pcr | TF | 26 | 26 | 1,989,538 | 99.81% | 12870.68 |
| YZ061 | YZ061 | 2021/7/31 | 2021/8/4 | II | Moderate | OPS | YZ | multiplex-pcr | Illumina | 24 | 25 | 382,249 | 99.61% | 1520.48 |
| YZ062 | YZ062 | 2021/7/31 | 2021/8/4 | II | Moderate | OPS | YZ | multiplex-pcr | TF | 16 | 14 | 5,822,047 | 99.84% | 37460.54 |
| YZ063 | YZ063 | 2021/7/31 | 2021/8/2 | II | Moderate | OPS | YZ | multiplex-pcr | BGI | 18 | 20 | 8,266,380 | 99.86% | 19679.56 |
| YZ064 | YZ064 | 2021/7/31 | 2021/8/2 | II | Mild | OPS | YZ | multiplex-pcr | BGI | 12 | 15 | 1,059,825 | 99.47% | 2518.665 |
| YZ065 | YZ065 | 2021/8/1 | 2021/8/4 | II | Moderate | OPS | YZ | multiplex-pcr | TF | 37 | 30 | 100,167 | 99.56% | 701.6162 |
| YZ066 | YZ066 | 2021/8/1 | 2021/8/2 | II | Mild | OPS | YZ | multiplex-pcr | BGI | 25 | 25 | 9,546,646 | 99.87% | 23531.25 |
| YZ067 | YZ067 | 2021/7/31 | 2021/8/2 | II | Moderate | OPS | YZ | multiplex-pcr | BGI | 14 | 18 | 8,243,515 | 99.94% | 19534.97 |
| YZ069 | YZ069 | 2021/7/31 | 2021/8/2 | II | Moderate | OPS | YZ | multiplex-pcr | BGI | 22 | 23 | 8,357,344 | 99.86% | 20053.69 |
| YZ070 | YZ070 | 2021/7/28 | 2021/8/2 | NE | Moderate | OPS | YZ | multiplex-pcr | Illumina | 20 | 20 | 2,417,562 | 99.94% | 9297.844 |
| YZ074 | YZ074 | 2021/7/29 | 2021/8/2 | NE | Moderate | OPS | YZ | multiplex-pcr | BGI | 32 | 31 | 1,327,834 | 99.75% | 3175.667 |
| YZ076 | YZ076 | 2021/7/30 | 2021/8/2 | II | Moderate | OPS | YZ | multiplex-pcr | BGI | 20 | 21 | 11,193,272 | 99.86% | 26883.59 |
| YZ077 | YZ077 | 2021/7/30 | 2021/8/4 | II | Moderate | OPS | YZ | multiplex-pcr | TF | 23 | 23.5 | 941,026 | 99.79% | 5562.444 |
| YZ079 | YZ079 | 2021/7/31 | 2021/8/4 | II | Moderate | OPS | YZ | multiplex-pcr | TF | 24.5 | 25 | 713,348 | 99.72% | 4244.41 |
| YZ080 | YZ080 | 2021/7/24 | 2021/8/2 | NE | Moderate | OPS | YZ | multiplex-pcr | BGI | 17 | 19 | 18,803,063 | 99.87% | 45081.26 |
| YZ081 | YZ081 | 2021/7/26 | 2021/8/2 | NE | Critical | OPS | YZ | multiplex-pcr | BGI | 12 | 14 | 7,317,739 | 99.94% | 17501.15 |
| YZ082 | YZ082 | 2021/7/28 | 2021/8/2 | NE | Severe | OPS | YZ | multiplex-pcr | Illumina | 37 | 27 | 5,224,634 | 99.94% | 20784.64 |
| YZ083 | YZ083 | 2021/7/29 | 2021/8/4 | II | Moderate | OPS | YZ | multiplex-pcr | TF | 28 | 27 | 2,795,146 | 99.83% | 19406.64 |
| YZ084 | YZ084 | 2021/7/31 | 2021/8/4 | II | Moderate | OPS | YZ | multiplex-pcr | TF | 14 | 14 | 3,967,031 | 99.89% | 25247.41 |
| YZ085 | YZ085 | 2021/8/1 | 2021/8/10 | II | Moderate | OPS | YZ | multiplex-pcr | Illumina | 25 | 26 | 126,134 | 99.51% | 509.782 |
| YZ086 | YZ086 | 2021/8/1 | 2021/8/4 | II | Moderate | OPS | YZ | multiplex-pcr | TF | 29.8 | 29.1 | 1,669,001 | 99.73% | 11025.46 |
| YZ087 | YZ087 | 2021/7/29 | 2021/8/2 | NE | Moderate | OPS | YZ | multiplex-pcr | BGI | 11 | 14 | 3,896,582 | 99.82% | 9265.278 |
| YZ089 | YZ089 | 2021/7/30 | 2021/8/4 | NE | Moderate | OPS | YZ | multiplex-pcr | TF | 11.6 | 15.6 | 1,036,643 | 99.78% | 6065.386 |
| YZ091 | YZ091 | 2021/8/1 | 2021/8/18 | II | Critical | OPS | YZ | multiplex-pcr | Illumina | 33 | 29 | 2,361,315 | 99.86% | 9001.676 |
| YZ092 | YZ092 | 2021/7/31 | 2021/8/4 | II | Mild | OPS | YZ | multiplex-pcr | TF | 24 | 24 | 2,527,051 | 99.77% | 17389.03 |
| YZ094 | YZ094 | 2021/8/1 | 2021/8/4 | II | Moderate | OPS | YZ | multiplex-pcr | TF | 22.8 | 25 | 5,262,546 | 99.77% | 36825.15 |
| YZ095 | YZ095 | 2021/7/27 | 2021/8/2 | NE | Moderate | OPS | YZ | multiplex-pcr | BGI | 15 | 15 | 8,457,881 | 99.86% | 20202.7 |
| YZ096 | YZ096 | 2021/7/31 | 2021/8/4 | II | Moderate | OPS | YZ | multiplex-pcr | TF | 28 | 28 | 2,439,837 | 99.68% | 14895.12 |
| YZ097 | YZ097 | 2021/7/31 | 2021/8/2 | II | Mild | OPS | YZ | multiplex-pcr | BGI | 31 | 32 | 868,847 | 99.79% | 2075.863 |
| YZ098 | YZ098 | 2021/7/31 | 2021/8/2 | II | Moderate | OPS | YZ | multiplex-pcr | BGI | 32 | 30 | 533,844 | 99.76% | 1278.872 |
| YZ099 | YZ099 | 2021/8/1 | 2021/8/4 | NE | Moderate | OPS | YZ | multiplex-pcr | TF | 26 | 26 | 3,620,921 | 99.77% | 23267.64 |
| YZ100 | YZ100 | 2021/8/1 | 2021/8/4 | II | Severe | OPS | YZ | multiplex-pcr | TF | 21.5 | 21 | 4,584,156 | 99.83% | 26154.21 |
| YZ102 | YZ102 | 2021/7/25 | 2021/8/2 | NE | Severe | OPS | YZ | multiplex-pcr | Illumina | 14 | 15 | 7,330,743 | 99.87% | 17581.64 |
| YZ103 | YZ103 | 2021/7/31 | 2021/8/2 | II | Severe | OPS | YZ | multiplex-pcr | Illumina | 11 | 13 | 4,579,104 | 99.85% | 10873.22 |
| YZ104 | YZ104 | 2021/7/31 | 2021/8/2 | II | Moderate | OPS | YZ | multiplex-pcr | Illumina | 24 | 24 | 8,290,978 | 99.87% | 19951.34 |
| YZ105 | YZ105 | 2021/7/28 | 2021/8/4 | NE | Critical | OPS | YZ | multiplex-pcr | Illumina | 21.7 | 21.9 | 2,408,982 | 99.94% | 9570.654 |
| YZ106 | YZ106 | 2021/8/2 | 2021/8/4 | II | Moderate | OPS | YZ | multiplex-pcr | TF | 28.7 | 29 | 534,516 | 99.56% | 3070.922 |
| YZ108 | YZ108 | 2021/8/1 | 2021/8/2 | II | Moderate | OPS | YZ | multiplex-pcr | BGI | 20 | 20 | 10,261,348 | 99.89% | 24982.77 |
| YZ109 | YZ109 | 2021/7/31 | 2021/8/4 | II | Moderate | OPS | YZ | multiplex-pcr | TF | 11 | 11 | 5,216,616 | 99.92% | 32975.43 |
| YZ110 | YZ110 | 2021/7/30 | 2021/8/2 | NE | Moderate | OPS | YZ | multiplex-pcr | BGI | 14 | 15 | 13,716,800 | 99.93% | 32766.8 |
| YZ112 | YZ112 | 2021/7/31 | 2021/8/2 | II | Moderate | OPS | YZ | multiplex-pcr | BGI | 22 | 23 | 15,651,848 | 99.87% | 38105.87 |
| YZ115 | YZ115 | 2021/7/31 | 2021/8/10 | II | Moderate | OPS | YZ | multiplex-pcr | Illumina | 25 | 25 | 326,364 | 99.58% | 1282.524 |
| YZ118 | YZ118 | 2021/8/2 | 2021/8/4 | II | Mild | OPS | YZ | multiplex-pcr | TF | 32.5 | 29 | 883,515 | 98.34% | 4288.538 |
| YZ121 | YZ121 | 2021/8/1 | 2021/8/4 | II | Mild | OPS | YZ | multiplex-pcr | Illumina | 27.8 | 27.8 | 677,644 | 99.84% | 2645.484 |
| YZ124 | YZ124 | 2021/8/1 | 2021/8/4 | II | Moderate | OPS | YZ | multiplex-pcr | TF | 32.7 | 29 | 1,820,038 | 99.38% | 7518.475 |
| YZ125 | YZ125 | 2021/7/29 | 2021/8/2 | NE | Mild | OPS | YZ | multiplex-pcr | Illumina | 24 | 24 | 2,693,751 | 99.94% | 10449.46 |
| YZ126 | YZ126 | 2021/7/30 | 2021/8/2 | II | Moderate | OPS | YZ | multiplex-pcr | BGI | 29 | 28 | 534,362 | 99.76% | 1323.141 |
| YZ128 | YZ128 | 2021/8/2 | 2021/8/10 | II | Mild | OPS | YZ | multiplex-pcr | TF | 23 | 24 | 2,725,696 | 99.77% | 18569.52 |
| YZ129 | YZ129 | 2021/8/2 | 2021/8/10 | II | Mild | OPS | YZ | multiplex-pcr | Illumina | 20 | 20 | 4,735,926 | 99.94% | 18633.52 |
| YZ131 | YZ131 | 2021/8/2 | 2021/8/19 | II | Mild | OPS | YZ | multiplex-pcr | Illumina | 15 | 15 | 11,921,156 | 99.94% | 47331.99 |
| YZ134 | YZ134 | 2021/8/2 | 2021/8/4 | II | Moderate | OPS | YZ | multiplex-pcr | TF | 25.7 | 25.4 | 2,613,281 | 99.82% | 11572.27 |
| YZ135 | YZ135 | 2021/8/3 | 2021/8/4 | II | Moderate | OPS | YZ | multiplex-pcr | TF | 25.4 | 24.2 | 2,113,210 | 99.84% | 11769.19 |
| YZ137 | YZ137 | 2021/8/3 | 2021/8/4 | II | Moderate | OPS | YZ | multiplex-pcr | TF | 24 | 23.4 | 3,481,957 | 99.83% | 21265.62 |
| YZ140 | YZ140 | 2021/8/2 | 2021/8/10 | II | Critical | OPS | YZ | multiplex-pcr | Illumina | 27 | 27 | 291,450 | 99.58% | 1163.023 |
| YZ143 | YZ143 | 2021/7/31 | 2021/8/10 | II | Critical | OPS | YZ | multiplex-pcr | Illumina | 30 | 30 | 355,622 | 98.97% | 1424.866 |
| YZ146 | YZ146 | 2021/8/2 | 2021/8/10 | II | Moderate | OPS | YZ | multiplex-pcr | Illumina | 29 | 30 | 342,842 | 99.62% | 1364.552 |
| YZ149 | YZ149 | 2021/8/1 | 2021/8/2 | II | Moderate | OPS | YZ | multiplex-pcr | BGI | 13 | 12 | 3,476,175 | 99.85% | 8265.455 |
| YZ155 | YZ155 | 2021/8/4 | 2021/8/18 | II | Moderate | OPS | YZ | multiplex-pcr | Illumina | 30 | 30 | 2,870,743 | 99.59% | 10956.42 |
| YZ156 | YZ156 | 2021/8/3 | 2021/8/4 | II | Moderate | OPS | YZ | multiplex-pcr | TF | 28.5 | 27 | 4,516,139 | 99.78% | 25690.52 |
| YZ159 | YZ159 | 2021/8/3 | 2021/8/11 | II | Moderate | NPS | YZ | multiplex-pcr | Illumina | 37 | 37 | 2,132,592 | 99.87% | 7965.675 |
| YZ160 | YZ160 | 2021/8/3 | 2021/8/11 | II | Moderate | NPS | YZ | multiplex-pcr | BGI | 35 | 35 | 47,442 | 99.15% | 156.0289 |
| YZ161 | YZ161 | 2021/8/2 | 2021/8/11 | II | Moderate | NPS | YZ | multiplex-pcr | Illumina | 25 | 28 | 2,338,350 | 99.88% | 9279.92 |
| YZ162 | YZ162 | 2021/8/3 | 2021/8/11 | II | Moderate | OPS | YZ | multiplex-pcr | Illumina | 23 | 24 | 14,624,517 | 99.93% | 56873.11 |
| YZ163 | YZ163 | 2021/8/1 | 2021/8/2 | II | Moderate | OPS | YZ | multiplex-pcr | BGI | 26 | 24 | 19,885,750 | 99.87% | 49195.98 |
| YZ168 | YZ168 | 2021/7/30 | 2021/8/6 | II | Mild | OPS | YZ | multiplex-pcr | Illumina | 30 | 31 | 1,766,729 | 99.78% | 7074.892 |
| YZ169 | YZ169 | 2021/8/4 | 2021/8/6 | II | Moderate | OPS | YZ | multiplex-pcr | Illumina | 31 | 33 | 711,115 | 99.70% | 2651.302 |
| YZ170 | YZ170 | 2021/7/30 | 2021/8/11 | II | Moderate | NPS | YZ | multiplex-pcr | Illumina | 27 | 29 | 1,816,118 | 99.91% | 7103.912 |
| YZ171 | YZ171 | 2021/8/4 | 2021/8/6 | II | Moderate | OPS | YZ | multiplex-pcr | Illumina | 37 | 35 | 13,927,942 | 99.89% | 52769.75 |
| YZ172 | YZ172 | 2021/8/3 | 2021/8/14 | II | Moderate | OPS | YZ | multiplex-pcr | Illumina | 36 | 37 | 2,395,738 | 98.56% | 8997.583 |
| YZ175 | YZ175 | 2021/8/4 | 2021/8/5 | II | Moderate | OPS | YZ | multiplex-pcr | Illumina | 27 | 29 | 1,858,767 | 99.88% | 7435.49 |
| YZ178 | YZ178 | 2021/8/3 | 2021/8/4 | II | Moderate | NPS | YZ | multiplex-pcr | TF | 25.5 | 24 | 2,195,424 | 99.85% | 9906.395 |
| YZ181 | YZ181 | 2021/8/1 | 2021/8/11 | II | Mild | OPS | YZ | multiplex-pcr | BGI | 33 | 33 | 175,226 | 99.89% | 570.4045 |
| YZ182 | YZ182 | 2021/7/29 | 2021/8/11 | NE | Moderate | NPS | YZ | multiplex-pcr | Illumina | 26 | 28 | 21,147,871 | 99.90% | 83632.72 |
| YZ187 | YZ187 | 2021/8/3 | 2021/8/11 | II | Moderate | NPS | YZ | multiplex-pcr | Illumina | 34 | 34 | 11,843,814 | 99.82% | 41502.75 |
| YZ188 | YZ188 | 2021/7/31 | 2021/8/11 | II | Severe | NPS | YZ | multiplex-pcr | Illumina | 32 | 32 | 6,387,839 | 99.82% | 23724.33 |
| YZ192 | YZ192 | 2021/7/31 | 2021/8/11 | II | Moderate | NPS | YZ | multiplex-pcr | Illumina | 28 | 28 | 40,771,872 | 99.87% | 156992.8 |
| YZ196 | YZ196 | 2021/8/2 | 2021/8/6 | II | Moderate | NPS | YZ | multiplex-pcr | Illumina | 37 | 39 | 520,905 | 98.99% | 1957.041 |
| YZ198 | YZ198 | 2021/7/26 | 2021/8/2 | NE | Critical | NPS | YZ | multiplex-pcr | BGI | 20 | 21 | 13,806,372 | 99.85% | 33115.98 |
| YZ200 | YZ200 | 2021/7/31 | 2021/8/2 | II | Severe | NPS | YZ | multiplex-pcr | BGI | 34 | 32 | 128,528 | 99.74% | 310.6324 |
| YZ202 | YZ202 | 2021/8/4 | 2021/8/6 | II | Moderate | NPS | YZ | multiplex-pcr | Illumina | 34 | 35 | 1,748,828 | 99.79% | 6504.53 |
| YZ204 | YZ204 | 2021/7/31 | 2021/8/6 | II | Severe | NPS | YZ | multiplex-pcr | Illumina | 38 | 37 | 28,064,559 | 99.94% | 107814.6 |
| YZ210 | YZ210 | 2021/8/4 | 2021/8/6 | II | Moderate | OPS | YZ | multiplex-pcr | Illumina | 26 | 26 | 1,057,775 | 99.56% | 3941.723 |
| YZ211 | YZ211 | 2021/8/1 | 2021/8/6 | II | Moderate | OPS | YZ | multiplex-pcr | Illumina | 33 | 34 | 1,008,907 | 99.36% | 3957.272 |
| YZ212 | YZ212 | 2021/8/4 | 2021/8/6 | II | Moderate | OPS | YZ | multiplex-pcr | Illumina | 28 | 27 | 1,902,567 | 99.71% | 7617.377 |
| YZ214 | YZ214 | 2021/8/5 | 2021/8/6 | II | Mild | OPS | YZ | multiplex-pcr | Illumina | 32 | 33 | 1,111,334 | 99.44% | 4294.378 |
| YZ215 | YZ215 | 2021/8/3 | 2021/8/6 | II | Moderate | OPS | YZ | multiplex-pcr | Illumina | 34 | 34 | 2,035,002 | 99.50% | 7912.416 |
| YZ224 | YZ224 | 2021/8/5 | 2021/8/11 | II | Moderate | OPS | YZ | multiplex-pcr | Illumina | 33 | 33 | 2,073,165 | 99.52% | 8037.804 |
| YZ227 | YZ227 | 2021/8/4 | 2021/9/23 | NE | Moderate | NPS | YZ | multiplex-pcr | Illumina | 32 | 33 | 2,045,507 | 99.74% | 6873.464 |
| YZ229 | YZ229 | 2021/7/30 | 2021/8/2 | NE | Critical | NPS | YZ | multiplex-pcr | BGI | 27 | 26 | 12,727,899 | 99.87% | 31022.64 |
| YZ230 | YZ230 | 2021/8/3 | 2021/8/11 | II | Severe | NPS | YZ | multiplex-pcr | Illumina | 30 | 30 | 8,289,250 | 99.88% | 32209.97 |
| YZ232 | YZ232 | 2021/8/5 | 2021/8/6 | II | Moderate | NPS | YZ | multiplex-pcr | Illumina | 34 | 34 | 1,143,673 | 99.83% | 4428.969 |
| YZ234 | YZ234 | 2021/8/4 | 2021/8/6 | II | Moderate | NPS | YZ | multiplex-pcr | Illumina | 27 | 24 | 981,941 | 99.76% | 3879.749 |
| YZ235 | YZ235 | 2021/8/5 | 2021/8/6 | II | Critical | NPS | YZ | multiplex-pcr | Illumina | 25 | 23 | 482,974 | 99.57% | 1908.759 |
| YZ236 | YZ236 | 2021/8/4 | 2021/8/19 | II | Moderate | NPS | YZ | multiplex-pcr | Illumina | 33 | 32 | 1,218,309 | 99.85% | 4509.005 |
| YZ240 | YZ240 | 2021/8/5 | 2021/8/19 | II | Moderate | NPS | YZ | multiplex-pcr | Illumina | 33 | 33 | 6,920,826 | 99.84% | 22568.44 |
| YZ242 | YZ242 | 2021/8/5 | 2021/8/11 | II | Mild | NPS | YZ | multiplex-pcr | Illumina | 24 | 24 | 23,152,212 | 99.94% | 92512.51 |
| YZ249 | YZ249 | 2021/8/4 | 2021/8/6 | II | Moderate | OPS | YZ | multiplex-pcr | Illumina | 22 | 21 | 1,351,732 | 99.56% | 5197.615 |
| YZ251 | YZ251 | 2021/8/5 | 2021/8/18 | II | Mild | OPS | YZ | multiplex-pcr | Illumina | 30 | 32 | 1,862,987 | 99.90% | 6984.405 |
| YZ254 | YZ254 | 2021/8/5 | 2021/8/11 | II | Severe | NPS | YZ | multiplex-pcr | Illumina | 31 | 31 | 10,806,436 | 99.93% | 40182.28 |
| YZ256 | YZ256 | 2021/8/4 | 2021/8/18 | II | Moderate | OPS | YZ | multiplex-pcr | Illumina | 33 | 34 | 295,292 | 99.24% | 1116.229 |
| YZ265 | YZ265 | 2021/8/5 | 2021/8/11 | II | Moderate | NPS | YZ | multiplex-pcr | BGI | 30 | 30 | 373,658 | 99.89% | 1246.48 |
| YZ266 | YZ266 | 2021/8/5 | 2021/8/11 | II | Moderate | NPS | YZ | multiplex-pcr | BGI | 18 | 18 | 352,353 | 99.94% | 1147.764 |
| YZ268 | YZ268 | 2021/8/5 | 2021/8/11 | II | Moderate | NPS | YZ | multiplex-pcr | BGI | 35 | 35 | 62,419 | 99.47% | 204.7944 |
| YZ271 | YZ271 | 2021/8/5 | 2021/8/19 | II | Mild | OPS | YZ | multiplex-pcr | Illumina | 31 | 31 | 3,604,361 | 99.87% | 13824.5 |
| YZ272 | YZ272 | 2021/8/5 | 8/18/2021 | II | Mild | OPS | YZ | multiplex-pcr | Illumina | 32 | 34 | 18,416,391 | 99.93% | 68807.29 |
| YZ275 | YZ275 | 2021/8/6 | 2021/8/7 | II | Moderate | NPS | YZ | multiplex-pcr | Illumina | 19 | 19 | 643,515 | 99.93% | 2149.275 |
| YZ278 | YZ278 | 2021/8/1 | 2021/8/19 | II | Moderate | NPS | YZ | multiplex-pcr | Illumina | 26 | 27 | 10,281,151 | 99.93% | 39609.08 |
| YZ279 | YZ279 | 2021/8/5 | 2021/8/11 | II | Moderate | NPS | YZ | multiplex-pcr | Illumina | 33 | 33 | 273,118 | 99.56% | 1024.564 |
| YZ280 | YZ280 | 2021/8/5 | 2021/8/19 | II | Moderate | NPS | YZ | multiplex-pcr | Illumina | 23 | 25 | 17,762,709 | 99.94% | 66302.55 |
| YZ281 | YZ281 | 2021/8/5 | 2021/8/18 | II | Moderate | NPS | YZ | multiplex-pcr | Illumina | 29 | 29 | 11,088,918 | 99.94% | 41678.39 |
| YZ282 | YZ282 | 2021/8/6 | 2021/8/19 | II | Moderate | NPS | YZ | multiplex-pcr | Illumina | 14 | 14 | 16,938,798 | 99.94% | 64715.86 |
| YZ283 | YZ283 | 2021/8/5 | 2021/8/19 | II | Moderate | NPS | YZ | multiplex-pcr | Illumina | 17 | 17 | 14,419,736 | 99.94% | 56103.2 |
| YZ284 | YZ284 | 2021/8/5 | 2021/8/18 | II | Moderate | NPS | YZ | multiplex-pcr | Illumina | 16 | 17 | 21,164,008 | 99.94% | 73816.79 |
| YZ285 | YZ285 | 2021/8/6 | 2021/8/11 | II | Moderate | NPS | YZ | multiplex-pcr | Illumina | 33 | 33 | 1,530,147 | 99.85% | 5789.582 |
| YZ286 | YZ286 | 2021/8/7 | 2021/8/19 | II | Moderate | NPS | YZ | multiplex-pcr | Illumina | 19 | 19 | 14,431,679 | 99.93% | 55292.96 |
| YZ288 | YZ288 | 2021/8/5 | 2021/8/11 | II | Severe | NPS | YZ | multiplex-pcr | Illumina | 32 | 32 | 2,394,309 | 99.83% | 9104.587 |
| YZ289 | YZ289 | 2021/8/5 | 2021/8/7 | II | Moderate | NPS | YZ | multiplex-pcr | Illumina | 31 | 28 | 684,518 | 99.68% | 2284.642 |
| YZ290 | YZ290 | 2021/8/6 | 2021/8/11 | II | Moderate | NPS | YZ | multiplex-pcr | Illumina | 27 | 27 | 3,446,589 | 99.68% | 12361.52 |
| YZ291 | YZ291 | 2021/8/6 | 2021/8/18 | II | Moderate | NPS | YZ | multiplex-pcr | Illumina | 29 | 29 | 2,274,406 | 99.59% | 8934.633 |
| YZ298 | YZ298 | 2021/8/6 | 2021/8/11 | II | Severe | NPS | YZ | multiplex-pcr | Illumina | 31 | 31 | 19,327,073 | 99.94% | 73727.15 |
| YZ303 | YZ303 | 2021/8/5 | 2021/8/11 | II | Moderate | NPS | YZ | multiplex-pcr | Illumina | 33 | 33 | 33,984,348 | 99.88% | 121622.1 |
| YZ304 | YZ304 | 2021/8/6 | 2021/8/11 | II | Moderate | NPS | YZ | multiplex-pcr | Illumina | 26 | 28 | 24,080,266 | 99.87% | 95047.11 |
| YZ307 | YZ307 | 2021/8/2 | 2021/8/19 | II | Moderate | NPS | YZ | multiplex-pcr | Illumina | 24 | 25 | 13,970,217 | 99.93% | 52657.07 |
| YZ308 | YZ308 | 2021/8/4 | 2021/8/11 | II | Moderate | NPS | YZ | multiplex-pcr | Illumina | 23 | 24 | 2,005,515 | 99.86% | 7927.65 |
| YZ309 | YZ309 | 2021/8/6 | 2021/8/11 | II | Moderate | NPS | YZ | multiplex-pcr | Illumina | 27 | 28 | 1,890,571 | 99.84% | 7555.75 |
| YZ310 | YZ310 | 2021/8/6 | 2021/8/11 | II | Moderate | NPS | YZ | multiplex-pcr | Illumina | 22 | 23 | 1,891,507 | 99.93% | 6243.864 |
| YZ311 | YZ311 | 2021/8/7 | 2021/8/18 | II | Moderate | NPS | YZ | multiplex-pcr | Illumina | 27 | 28 | 6,626,249 | 99.88% | 25058.76 |
| YZ315 | YZ315 | 2021/8/5 | 2021/8/11 | II | Moderate | OPS | YZ | multiplex-pcr | Illumina | 28 | 28.2 | 5,013,865 | 99.90% | 18425.42 |
| YZ319 | YZ319 | 2021/8/7 | 2021/8/8 | II | Mild | NPS | YZ | multiplex-pcr | Illumina | 27 | 26 | 193,330 | 99.74% | 784.58 |
| YZ323 | YZ323 | 2021/8/7 | 2021/8/8 | II | Moderate | NPS | YZ | multiplex-pcr | Illumina | 26 | 26 | 369,023 | 99.74% | 1455.258 |
| YZ326 | YZ326 | 2021/8/7 | 2021/8/11 | II | Moderate | NPS | YZ | multiplex-pcr | Illumina | 29 | 30 | 3,056,235 | 99.93% | 11225.91 |
| YZ328 | YZ328 | 2021/8/7 | 2021/8/11 | II | Moderate | NPS | YZ | multiplex-pcr | Illumina | 21 | 22 | 1,731,558 | 99.94% | 6924.178 |
| YZ329 | YZ329 | 2021/8/7 | 2021/8/11 | II | Moderate | NPS | YZ | multiplex-pcr | Illumina | 32 | 35 | 350,607 | 99.75% | 1320.504 |
| YZ330 | YZ330 | 2021/8/4 | 2021/8/19 | II | Moderate | NPS | YZ | multiplex-pcr | Illumina | 29 | 29 | 16,093,247 | 99.86% | 60984.61 |
| YZ331 | YZ331 | 2021/8/2 | 2021/8/11 | II | Moderate | NPS | YZ | multiplex-pcr | Illumina | 30 | 30 | 1,533,060 | 99.86% | 5568.201 |
| YZ334 | YZ334 | 2021/8/7 | 2021/8/8 | II | Moderate | NPS | YZ | multiplex-pcr | Illumina | 25 | 25 | 189,594 | 99.56% | 759.6693 |
| YZ338 | YZ338 | 2021/8/4 | 2021/8/11 | II | Moderate | NPS | YZ | multiplex-pcr | Illumina | 20 | 22 | 31,980,006 | 99.94% | 124808.9 |
| YZ339 | YZ339 | 2021/8/1 | 2021/8/11 | II | Mild | NPS | YZ | multiplex-pcr | Illumina | 32 | 32 | 7,882,454 | 99.82% | 29740.9 |
| YZ340 | YZ340 | 2021/8/7 | 2021/8/8 | II | Mild | NPS | YZ | multiplex-pcr | Illumina | 22 | 20 | 576,961 | 99.77% | 2322.174 |
| YZ341 | YZ341 | 2021/8/8 | 2021/8/8 | CI | Moderate | NPS | YZ | multiplex-pcr | Illumina | 22 | 21 | 607,944 | 99.58% | 2444.163 |
| YZ342 | YZ342 | 2021/8/5 | 2021/8/18 | II | Moderate | NPS | YZ | multiplex-pcr | Illumina | 30 | 30 | 7,351,180 | 99.88% | 27594.85 |
| YZ343 | YZ343 | 2021/7/25 | 2021/8/2 | NE | Moderate | OPS | YZ | multiplex-pcr | BGI | 32 | 30 | 395,850 | 99.83% | 954.9447 |
| YZ345 | YZ345 | 2021/8/7 | 2021/8/9 | II | Moderate | NPS | YZ | multiplex-pcr | Illumina | 34 | 38 | 1,336,398 | 99.94% | 5253.582 |
| YZ348 | YZ348 | 2021/8/7 | 2021/8/11 | II | Moderate | NPS | YZ | multiplex-pcr | Illumina | 20 | 20 | 2,809,689 | 99.94% | 11277.17 |
| YZ350 | YZ350 | 2021/8/7 | 2021/8/11 | II | Moderate | NPS | YZ | multiplex-pcr | Illumina | 17 | 19 | 16,273,985 | 99.94% | 64186.88 |
| YZ351 | YZ351 | 2021/8/8 | 2021/8/18 | CI | Moderate | NPS | YZ | multiplex-pcr | Illumina | 33 | 34 | 2,479,611 | 99.07% | 9487.248 |
| YZ352 | YZ352 | 2021/8/8 | 2021/8/11 | CI | Mild | NPS | YZ | multiplex-pcr | Illumina | 30 | 30 | 30,167,034 | 99.85% | 115703.4 |
| YZ353 | YZ353 | 2021/8/6 | 2021/8/9 | II | Moderate | NPS | YZ | multiplex-pcr | Illumina | 28 | 28 | 2,774,725 | 99.94% | 11161.65 |
| YZ354 | YZ354 | 2021/8/7 | 2021/8/9 | II | Moderate | NPS | YZ | multiplex-pcr | Illumina | 34 | 34 | 2,138,256 | 98.99% | 7975.334 |
| YZ355 | YZ355 | 2021/8/6 | 2021/8/11 | II | Moderate | NPS | YZ | multiplex-pcr | Illumina | 17 | 20 | 23,935,317 | 99.94% | 91840.22 |
| YZ356 | YZ356 | 2021/8/7 | 2021/8/9 | II | Mild | NPS | YZ | multiplex-pcr | Illumina | 32 | 32 | 5,717,220 | 99.90% | 19674.22 |
| YZ357 | YZ357 | 2021/8/4 | 2021/8/11 | II | Mild | OPS | YZ | multiplex-pcr | Illumina | 32 | 33 | 1,362,037 | 99.81% | 5324.623 |
| YZ358 | YZ358 | 2021/8/6 | 2021/8/9 | II | Moderate | OPS | YZ | multiplex-pcr | Illumina | 27 | 27 | 3,313,288 | 99.92% | 13348.19 |
| YZ360 | YZ360 | 2021/8/7 | 2021/8/9 | II | Moderate | OPS | YZ | multiplex-pcr | Illumina | 30 | 32 | 1,670,497 | 99.82% | 5663.923 |
| YZ362 | YZ362 | 2021/8/5 | 2021/8/11 | II | Moderate | NPS | YZ | multiplex-pcr | Illumina | 24 | 24 | 30,911,678 | 99.99% | 123339.4 |
| YZ363 | YZ363 | 2021/8/4 | 2021/8/11 | II | Critical | NPS | YZ | multiplex-pcr | Illumina | 20 | 20 | 21,990,916 | 99.94% | 84499.94 |
| YZ364 | YZ364 | 2021/8/7 | 2021/8/11 | II | Moderate | NPS | YZ | multiplex-pcr | Illumina | 30 | 31 | 2,473,670 | 99.93% | 9687.334 |
| YZ365 | YZ365 | 2021/8/7 | 2021/8/11 | II | Moderate | NPS | YZ | multiplex-pcr | Illumina | 21 | 23 | 1,407,252 | 99.90% | 5587.949 |
| YZ366 | YZ366 | 2021/8/7 | 2021/8/9 | II | Moderate | NPS | YZ | multiplex-pcr | Illumina | 30 | 30 | 2,858,406 | 99.74% | 11499.21 |
| YZ367 | YZ367 | 2021/8/7 | 2021/8/9 | II | Mild | OPS | YZ | multiplex-pcr | Illumina | 30 | 30 | 3,016,439 | 99.93% | 12120.94 |
| YZ368 | YZ368 | 2021/8/8 | 2021/8/9 | CI | Moderate | NPS | YZ | multiplex-pcr | Illumina | 33 | 33 | 3,379,807 | 99.83% | 13612.13 |
| YZ374 | YZ374 | 2021/8/7 | 2021/8/11 | II | Mild | OPS | YZ | multiplex-pcr | Illumina | 32 | 33 | 3,008,852 | 99.86% | 11601.56 |
| YZ375 | YZ375 | 2021/8/6 | 2021/8/11 | II | Moderate | NPS | YZ | multiplex-pcr | Illumina | 24 | 24 | 37,152,195 | 99.94% | 140957.5 |
| YZ376 | YZ376 | 2021/8/7 | 2021/8/12 | II | Mild | OPS | YZ | multiplex-pcr | Illumina | 26 | 26 | 1,456,716 | 99.29% | 5837.734 |
| YZ378 | YZ378 | 2021/8/8 | 2021/8/11 | CI | Moderate | OPS | YZ | multiplex-pcr | Illumina | 36 | 35 | 1,734,739 | 99.72% | 6971.058 |
| YZ380 | YZ380 | 2021/8/4 | 2021/8/11 | II | Moderate | NPS | YZ | multiplex-pcr | Illumina | 20 | 20 | 19,612,173 | 99.94% | 75911.87 |
| YZ381 | YZ381 | 2021/8/8 | 2021/8/11 | CI | Mild | OPS | YZ | multiplex-pcr | Illumina | 27 | 28 | 1,963,325 | 99.90% | 7867.678 |
| YZ383 | YZ383 | 2021/8/4 | 2021/8/11 | II | Moderate | NPS | YZ | multiplex-pcr | Illumina | 24 | 24 | 23,052,751 | 99.94% | 91010.4 |
| YZ385 | YZ385 | 2021/8/8 | 2021/8/11 | CI | Moderate | NPS | YZ | multiplex-pcr | Illumina | 19 | 20 | 14,918,001 | 99.94% | 58514.6 |
| YZ386 | YZ386 | 2021/8/8 | 2021/8/11 | CI | Severe | NPS | YZ | multiplex-pcr | Illumina | 26 | 28 | 24,948,522 | 99.94% | 97426.32 |
| YZ387 | YZ387 | 2021/8/7 | 2021/8/12 | II | Moderate | OPS | YZ | multiplex-pcr | Illumina | 29 | 28 | 1,225,594 | 99.13% | 4899.398 |
| YZ388 | YZ388 | 2021/8/8 | 2021/8/10 | CI | Mild | OPS | YZ | multiplex-pcr | TF | 29 | 28 | 763,636 | 99.77% | 4627.807 |
| YZ391 | YZ391 | 2021/8/8 | 2021/8/11 | CI | Moderate | NPS | YZ | multiplex-pcr | Illumina | 16 | 18 | 17,871,746 | 99.94% | 70134.84 |
| YZ392 | YZ392 | 2021/8/5 | 2021/8/11 | II | Moderate | NPS | YZ | multiplex-pcr | Illumina | 26 | 27 | 20,039,407 | 99.94% | 79100.37 |
| YZ393 | YZ393 | 2021/8/8 | 2021/8/11 | CI | Moderate | NPS | YZ | multiplex-pcr | Illumina | 24 | 26 | 26,345,467 | 99.84% | 103677.7 |
| YZ394 | YZ394 | 2021/8/2 | 2021/8/11 | II | Moderate | NPS | YZ | multiplex-pcr | Illumina | 32 | 33 | 38,343,842 | 99.88% | 134896.5 |
| YZ395 | YZ395 | 2021/8/9 | 2021/8/11 | CI | Mild | NPS | YZ | multiplex-pcr | Illumina | 22 | 25 | 22,988,304 | 99.93% | 88592.28 |
| YZ396 | YZ396 | 2021/8/8 | 2021/8/11 | CI | Severe | NPS | YZ | multiplex-pcr | Illumina | 24 | 25 | 23,320,978 | 99.86% | 87800.49 |
| YZ397 | YZ397 | 2021/8/8 | 2021/8/10 | CI | Moderate | OPS | YZ | multiplex-pcr | TF | 20 | 20 | 2,652,447 | 99.91% | 17511.01 |
| YZ398 | YZ398 | 2021/8/8 | 2021/8/11 | CI | Moderate | OPS | YZ | multiplex-pcr | Illumina | 30 | 31 | 1,515,258 | 99.93% | 5865.973 |
| YZ399 | YZ399 | 2021/8/7 | 2021/8/10 | II | Moderate | OPS | YZ | multiplex-pcr | TF | 21 | 20 | 2,529,031 | 99.89% | 16709.9 |
| YZ400 | YZ400 | 2021/8/8 | 2021/8/10 | CI | Moderate | OPS | YZ | multiplex-pcr | TF | 20 | 20 | 2,231,102 | 99.87% | 14778.87 |
| YZ401 | YZ401 | 2021/8/2 | 2021/8/10 | II | Moderate | OPS | YZ | multiplex-pcr | TF | 30 | 30 | 357,172 | 99.77% | 2278.9 |
| YZ402 | YZ402 | 2021/8/9 | 2021/8/18 | CI | Moderate | OPS | YZ | multiplex-pcr | Illumina | 27 | 29 | 4,306,711 | 99.93% | 15569.48 |
| YZ403 | YZ403 | 2021/8/6 | 2021/8/10 | II | Moderate | OPS | YZ | multiplex-pcr | TF | 25 | 25 | 1,156,317 | 99.77% | 7172.973 |
| YZ404 | YZ404 | 2021/8/10 | 2021/8/11 | CI | Moderate | OPS | YZ | multiplex-pcr | BGI | 25 | 25 | 1,224,800 | 99.90% | 4085.871 |
| YZ405 | YZ405 | 2021/8/9 | 2021/8/11 | CI | Mild | OPS | YZ | multiplex-pcr | BGI | 26 | 26 | 1,453,957 | 99.89% | 4852.018 |
| YZ406 | YZ406 | 2021/8/7 | 2021/8/11 | II | Moderate | OPS | YZ | multiplex-pcr | BGI | 27 | 27 | 1,214,804 | 99.89% | 4052.508 |
| YZ407 | YZ407 | 2021/8/4 | 2021/8/10 | II | Mild | NPS | YZ | multiplex-pcr | TF | 32 | 30 | 412,336 | 99.77% | 2489.916 |
| YZ408 | YZ408 | 2021/8/10 | 2021/8/11 | CI | Severe | NPS | YZ | multiplex-pcr | Illumina | 16 | 18 | 28,494,535 | 99.94% | 112487.6 |
| YZ409 | YZ409 | 2021/8/8 | 2021/8/10 | CI | Moderate | OPS | YZ | multiplex-pcr | TF | 27 | 25 | 2,508,870 | 99.81% | 15970.54 |
| YZ410 | YZ410 | 2021/8/7 | 2021/8/10 | II | Moderate | OPS | YZ | multiplex-pcr | TF | 25 | 24 | 1,824,349 | 99.85% | 12249.53 |
| YZ411 | YZ411 | 2021/8/5 | 2021/8/11 | II | Moderate | NPS | YZ | multiplex-pcr | BGI | 31 | 31 | 617,533 | 99.89% | 2060.022 |
| YZ412 | YZ412 | 2021/8/8 | 2021/8/10 | CI | Moderate | NPS | YZ | multiplex-pcr | TF | 24 | 24 | 1,734,326 | 99.84% | 11104.85 |
| YZ413 | YZ413 | 2021/8/6 | 2021/8/10 | II | Moderate | NPS | YZ | multiplex-pcr | TF | 32 | 30 | 214,632 | 99.77% | 1359.505 |
| YZ414 | YZ414 | 2021/8/9 | 2021/8/10 | CI | Mild | NPS | YZ | multiplex-pcr | TF | 27 | 27 | 1,667,594 | 99.78% | 10742.46 |
| YZ415 | YZ415 | 2021/8/9 | 2021/8/10 | CI | Mild | NPS | YZ | multiplex-pcr | TF | 28 | 28 | 1,153,930 | 99.82% | 7174.557 |
| YZ416 | YZ416 | 2021/8/9 | 2021/8/10 | CI | Moderate | NPS | YZ | multiplex-pcr | TF | 20 | 22 | 2,983,963 | 99.82% | 20091.1 |
| YZ417 | YZ417 | 2021/8/8 | 2021/8/10 | CI | Mild | NPS | YZ | multiplex-pcr | TF | 18 | 20 | 1,775,859 | 99.83% | 11657.68 |
| YZ418 | YZ418 | 2021/8/8 | 2021/8/11 | CI | Mild | NPS | YZ | multiplex-pcr | Illumina | 18 | 19 | 1,899,110 | 99.94% | 7672.136 |
| YZ419 | YZ419 | 2021/8/9 | 2021/8/11 | CI | Mild | NPS | YZ | multiplex-pcr | Illumina | 33 | 34 | 507,801 | 98.89% | 2010.902 |
| YZ420 | YZ420 | 2021/8/7 | 2021/8/11 | II | Moderate | NPS | YZ | multiplex-pcr | Illumina | 23 | 25 | 1,942,947 | 99.84% | 7820.37 |
| YZ422 | YZ422 | 2021/8/4 | 2021/8/8 | II | Moderate | NPS | YZ | multiplex-pcr | Illumina | 30 | 29 | 102,863 | 99.47% | 417.3331 |
| YZ424 | YZ424 | 2021/8/9 | 2021/8/11 | CI | Moderate | NPS | YZ | multiplex-pcr | Illumina | 20 | 21 | 2,281,297 | 99.93% | 8413.484 |
| YZ425 | YZ425 | 2021/8/9 | 2021/8/10 | CI | Moderate | NPS | YZ | multiplex-pcr | TF | 31 | 32 | 2,101,102 | 99.78% | 14234.28 |
| YZ426 | YZ426 | 2021/8/8 | 2021/8/10 | CI | Moderate | OPS | YZ | multiplex-pcr | TF | 30 | 29 | 2,229,780 | 99.81% | 14772.75 |
| YZ427 | YZ427 | 2021/8/9 | 2021/8/10 | CI | Moderate | NPS | YZ | multiplex-pcr | Illumina | 35 | 30 | 3,730,192 | 99.88% | 13639.17 |
| YZ428 | YZ428 | 2021/8/10 | 2021/8/10 | CI | Mild | NPS | YZ | multiplex-pcr | Illumina | 24 | 24 | 1,656,503 | 99.81% | 6658.233 |
| YZ429 | YZ429 | 2021/8/9 | 2021/8/10 | CI | Moderate | NPS | YZ | multiplex-pcr | Illumina | 35 | 34 | 3,354,830 | 99.94% | 12838.89 |
| YZ430 | YZ430 | 2021/8/9 | 2021/8/10 | CI | Mild | NPS | YZ | multiplex-pcr | TF | 26 | 26 | 1,974,665 | 99.85% | 13287.63 |
| YZ431 | YZ431 | 2021/8/9 | 2021/8/10 | CI | Moderate | NPS | YZ | multiplex-pcr | TF | 31 | 30 | 2,288,925 | 99.79% | 15475.22 |
| YZ433 | YZ433 | 2021/8/7 | 2021/8/10 | II | Moderate | NPS | YZ | multiplex-pcr | TF | 22 | 24 | 2,458,768 | 99.82% | 15928.28 |
| YZ434 | YZ434 | 2021/8/9 | 2021/8/10 | CI | Moderate | NPS | YZ | multiplex-pcr | TF | 25 | 25 | 2,354,189 | 99.75% | 15823.29 |
| YZ435 | YZ435 | 2021/8/9 | 2021/8/10 | CI | Severe | OPS | YZ | multiplex-pcr | TF | 25 | 26 | 2,017,316 | 99.77% | 13390.97 |
| YZ436 | YZ436 | 2021/8/7 | 2021/8/10 | II | Mild | NPS | YZ | multiplex-pcr | Illumina | 30 | 30 | 2,467,858 | 99.91% | 9915.299 |
| YZ438 | YZ438 | 2021/8/4 | 2021/8/18 | II | Moderate | NPS | YZ | multiplex-pcr | Illumina | 24 | 25 | 1,854,648 | 99.59% | 6975.67 |
| YZ439 | YZ439 | 2021/8/9 | 2021/8/18 | CI | Moderate | NPS | YZ | multiplex-pcr | Illumina | 30 | 30 | 697,645 | 98.89% | 2372.728 |
| YZ440 | YZ440 | 2021/8/7 | 2021/8/18 | II | Severe | NPS | YZ | multiplex-pcr | Illumina | 23 | 24 | 9,925,468 | 99.94% | 38033.45 |
| YZ441 | YZ441 | 2021/8/5 | 2021/8/10 | II | Moderate | NPS | YZ | multiplex-pcr | TF | 30 | 30 | 2,011,151 | 99.78% | 13634.75 |
| YZ442 | YZ442 | 2021/8/7 | 2021/8/10 | II | Moderate | NPS | YZ | multiplex-pcr | TF | 25 | 26 | 2,190,698 | 99.78% | 14740.3 |
| YZ443 | YZ443 | 2021/8/4 | 2021/8/10 | II | Moderate | NPS | YZ | multiplex-pcr | TF | 21 | 24 | 2,462,431 | 99.79% | 16727.03 |
| YZ444 | YZ444 | 2021/8/9 | 2021/8/12 | CI | Moderate | NPS | YZ | multiplex-pcr | Illumina | 31 | 31 | 2,265,480 | 99.58% | 9028.512 |
| YZ446 | YZ446 | 2021/8/6 | 2021/8/11 | II | Moderate | OPS | YZ | multiplex-pcr | Illumina | 24 | 25 | 2,061,177 | 99.86% | 8270.156 |
| YZ448 | YZ448 | 2021/8/10 | 2021/8/11 | CI | Moderate | NPS | YZ | multiplex-pcr | Illumina | 25 | 26 | 25,731,971 | 99.87% | 98132.75 |
| YZ450 | YZ450 | 2021/8/9 | 2021/8/11 | CI | Moderate | NPS | YZ | multiplex-pcr | Illumina | 18 | 20 | 17,833,578 | 99.94% | 71029.37 |
| YZ451 | YZ451 | 2021/8/9 | 2021/8/11 | CI | Moderate | NPS | YZ | multiplex-pcr | Illumina | 18 | 20 | 21,514,559 | 99.94% | 85342.05 |
| YZ452 | YZ452 | 2021/8/8 | 2021/8/10 | CI | Mild | NPS | YZ | multiplex-pcr | TF | 27 | 27 | 1,573,036 | 99.80% | 10191.64 |
| YZ453 | YZ453 | 2021/8/5 | 2021/8/11 | II | Moderate | NPS | YZ | multiplex-pcr | Illumina | 24 | 26 | 19,218,774 | 99.79% | 74832.61 |
| YZ454 | YZ454 | 2021/8/10 | 2021/8/11 | CI | Moderate | NPS | YZ | multiplex-pcr | Illumina | 32 | 33 | 16,456,867 | 99.83% | 60148.29 |
| YZ455 | YZ455 | 2021/8/10 | 2021/8/11 | CI | Mild | NPS | YZ | multiplex-pcr | Illumina | 20 | 20 | 20,465,691 | 99.94% | 81403.3 |
| YZ456 | YZ456 | 2021/8/10 | 2021/8/11 | CI | Moderate | NPS | YZ | multiplex-pcr | Illumina | 28 | 29 | 641,400 | 99.55% | 2597.14 |
| YZ458 | YZ458 | 2021/8/9 | 2021/8/11 | CI | Mild | NPS | YZ | multiplex-pcr | Illumina | 18 | 20 | 1,880,124 | 99.94% | 7588.175 |
| YZ459 | YZ459 | 2021/8/10 | 2021/8/12 | CI | Moderate | OPS | YZ | multiplex-pcr | Illumina | 0 | 33 | 3,311,557 | 99.77% | 12476.32 |
| YZ460 | YZ460 | 2021/8/10 | 2021/8/11 | CI | Mild | NPS | YZ | multiplex-pcr | Illumina | 29 | 28 | 802,102 | 99.57% | 3080.047 |
| YZ461 | YZ461 | 2021/8/9 | 2021/8/11 | CI | Moderate | NPS | YZ | multiplex-pcr | Illumina | 31 | 27 | 388,093 | 99.48% | 1579.776 |
| YZ462 | YZ462 | 2021/8/11 | 2021/8/19 | CI | Moderate | OPS | YZ | multiplex-pcr | Illumina | 28 | 29 | 8,306,466 | 99.24% | 28682.06 |
| YZ463 | YZ463 | 2021/8/11 | 2021/8/11 | CI | Moderate | NPS | YZ | multiplex-pcr | Illumina | 33 | 33 | 818,577 | 99.60% | 3196.083 |
| YZ465 | YZ465 | 2021/8/5 | 2021/8/12 | II | Moderate | OPS | YZ | multiplex-pcr | BGI | 29 | 30 | 5,972,548 | 99.90% | 19055.78 |
| YZ466 | YZ466 | 2021/8/8 | 2021/8/12 | CI | Moderate | OPS | YZ | multiplex-pcr | BGI | 24 | 25 | 14,110,668 | 99.94% | 44831.1 |
| YZ467 | YZ467 | 2021/8/10 | 2021/8/12 | CI | Mild | OPS | YZ | multiplex-pcr | BGI | 24 | 25 | 11,518,392 | 99.94% | 36399.24 |
| YZ468 | YZ468 | 2021/8/10 | 2021/8/18 | CI | Moderate | OPS | YZ | multiplex-pcr | Illumina | 23 | 24 | 10,467,360 | 99.94% | 40308.7 |
| YZ469 | YZ469 | 2021/8/10 | 2021/8/12 | CI | Mild | OPS | YZ | multiplex-pcr | Illumina | 28 | 27 | 1,136,282 | 99.60% | 4600.826 |
| YZ470 | YZ470 | 2021/8/10 | 2021/8/12 | CI | Moderate | NPS | YZ | multiplex-pcr | Illumina | 31 | 30 | 1,097,410 | 99.58% | 4439.936 |
| YZ471 | YZ471 | 2021/8/10 | 2021/8/12 | CI | Mild | NPS | YZ | multiplex-pcr | Illumina | 29 | 28 | 1,287,959 | 99.45% | 5133.955 |
| YZ472 | YZ472 | 2021/8/10 | 2021/8/11 | CI | Mild | OPS | YZ | multiplex-pcr | Illumina | 31 | 32 | 2,297,388 | 99.78% | 9165.181 |
| YZ473 | YZ473 | 2021/8/10 | 2021/8/12 | CI | Mild | OPS | YZ | multiplex-pcr | Illumina | 36 | 35 | 1,794,043 | 99.86% | 7218.252 |
| YZ475 | YZ475 | 2021/8/10 | 2021/8/11 | CI | Mild | OPS | YZ | multiplex-pcr | Illumina | 30 | 30 | 762,025 | 99.57% | 2980.38 |
| YZ478 | YZ478 | 2021/8/7 | 2021/8/11 | II | Moderate | NPS | YZ | multiplex-pcr | Illumina | 22 | 22 | 24,686,913 | 99.94% | 95555.85 |
| YZ480 | YZ480 | 2021/8/10 | 2021/8/12 | CI | Moderate | OPS | YZ | multiplex-pcr | BGI | 22 | 24 | 13,775,207 | 99.94% | 43777.45 |
| YZ481 | YZ481 | 2021/8/10 | 2021/8/12 | CI | Severe | OPS | YZ | multiplex-pcr | BGI | 30 | 30 | 184,753 | 99.72% | 593.9336 |
| YZ484 | YZ484 | 2021/8/11 | 2021/8/18 | CI | Moderate | OPS | YZ | multiplex-pcr | Illumina | 30 | 31 | 6,065,307 | 99.72% | 23073.52 |
| YZ485 | YZ485 | 2021/8/10 | 2021/8/12 | CI | Mild | NPS | YZ | multiplex-pcr | Illumina | 33 | 34 | 26,732,537 | 99.93% | 105802.9 |
| YZ486 | YZ486 | 2021/8/10 | 2021/8/18 | CI | Moderate | OPS | YZ | multiplex-pcr | Illumina | 25 | 25 | 6,361,492 | 99.89% | 24114.53 |
| YZ488 | YZ488 | 2021/8/10 | 2021/8/18 | CI | Moderate | OPS | YZ | multiplex-pcr | Illumina | 23 | 24 | 11,791,744 | 99.94% | 44919.96 |
| YZ489 | YZ489 | 2021/8/11 | 2021/8/18 | CI | Moderate | OPS | YZ | multiplex-pcr | Illumina | 25 | 25 | 6,090,301 | 99.94% | 23121.42 |
| YZ492 | YZ492 | 2021/8/10 | 2021/8/12 | CI | Moderate | OPS | YZ | multiplex-pcr | Illumina | 30 | 30 | 2,829,372 | 99.63% | 11461.95 |
| YZ494 | YZ494 | 2021/8/9 | 2021/8/12 | CI | Moderate | OPS | YZ | multiplex-pcr | Illumina | 19 | 20 | 1,579,525 | 99.61% | 6290.944 |
| YZ497 | YZ497 | 2021/8/11 | 2021/8/12 | CI | Moderate | OPS | YZ | multiplex-pcr | Illumina | 20 | 21 | 1,100,006 | 99.60% | 4457.015 |
| YZ498 | YZ498 | 2021/8/6 | 2021/8/18 | II | Mild | OPS | YZ | multiplex-pcr | Illumina | 28 | 29 | 10,672,340 | 99.93% | 41085.47 |
| YZ499 | YZ499 | 2021/8/12 | 2021/8/13 | CI | Moderate | OPS | YZ | multiplex-pcr | Illumina | 26 | 27 | 1,679,771 | 99.86% | 6713.408 |
| YZ500 | YZ500 | 2021/8/11 | 2021/8/18 | CI | Mild | OPS | YZ | multiplex-pcr | Illumina | 24 | 25 | 10,123,903 | 99.91% | 38797.16 |
| YZ504 | YZ504 | 2021/8/10 | 2021/8/18 | CI | Mild | OPS | YZ | multiplex-pcr | Illumina | 29 | 29 | 1,131,290 | 99.45% | 3987.386 |
| YZ505 | YZ505 | 2021/8/12 | 2021/8/18 | CI | Mild | OPS | YZ | multiplex-pcr | Illumina | 25 | 26 | 399,825 | 98.99% | 1495.327 |
| YZ506 | YZ506 | 2021/8/11 | 2021/8/13 | CI | Mild | OPS | YZ | multiplex-pcr | Illumina | 25 | 32 | 358,592 | 99.02% | 1394.056 |
| YZ507 | YZ507 | 2021/8/11 | 2021/8/18 | CI | Moderate | OPS | YZ | multiplex-pcr | Illumina | 32 | 33 | 4,194,722 | 99.93% | 16049.52 |
| YZ509 | YZ509 | 2021/8/10 | 2021/8/18 | CI | Mild | OPS | YZ | multiplex-pcr | Illumina | 32 | 33 | 2,897,692 | 99.86% | 10708 |
| YZ510 | YZ510 | 2021/8/10 | 2021/8/18 | CI | Moderate | OPS | YZ | multiplex-pcr | Illumina | 27 | 27 | 8,878,686 | 99.94% | 33662.13 |
| YZ512 | YZ512 | 2021/8/11 | 2021/8/13 | CI | Moderate | OPS | YZ | multiplex-pcr | Illumina | 23 | 27 | 1,423,963 | 99.88% | 5715.885 |
| YZ514 | YZ514 | 2021/8/12 | 2021/8/18 | CI | Moderate | OPS | YZ | multiplex-pcr | Illumina | 25 | 25 | 2,730,071 | 99.64% | 10381.09 |
| YZ515 | YZ515 | 2021/8/12 | 2021/8/16 | CI | Moderate | OPS | YZ | multiplex-pcr | Illumina | 31 | 34 | 701,680 | 99.75% | 2625.356 |
| YZ516 | YZ516 | 2021/8/9 | 2021/8/16 | CI | Moderate | OPS | YZ | multiplex-pcr | Illumina | 25 | 26 | 1,578,775 | 99.83% | 6312.204 |
| YZ517 | YZ517 | 2021/8/12 | 2021/8/16 | CI | Mild | OPS | YZ | multiplex-pcr | Illumina | 30 | 28 | 1,689,086 | 99.84% | 6751.423 |
| YZ518 | YZ518 | 2021/8/12 | 2021/8/18 | CI | Moderate | NPS | YZ | multiplex-pcr | Illumina | 18 | 19 | 10,403,785 | 99.93% | 36565.65 |
| YZ519 | YZ519 | 2021/8/12 | 2021/8/18 | CI | Moderate | OPS | YZ | multiplex-pcr | Illumina | 23 | 24 | 1,383,281 | 99.88% | 4847.055 |
| YZ522 | YZ522 | 2021/8/12 | 2021/8/13 | CI | Moderate | OPS | YZ | multiplex-pcr | Illumina | 23 | 24 | 980,260 | 99.76% | 3782.601 |
| YZ523 | YZ523 | 2021/8/12 | 2021/8/18 | CI | Moderate | OPS | YZ | multiplex-pcr | Illumina | 28 | 29 | 889,943 | 99.59% | 3267.013 |
| YZ524 | YZ524 | 2021/8/13 | 2021/8/14 | CI | Moderate | OPS | YZ | multiplex-pcr | Illumina | 24 | 25 | 1,437,113 | 99.90% | 5781.894 |
| YZ525 | YZ525 | 2021/8/12 | 2021/8/18 | CI | Moderate | OPS | YZ | multiplex-pcr | Illumina | 19 | 20 | 11,534,996 | 99.91% | 44240.5 |
| YZ526 | YZ526 | 2021/8/10 | 2021/8/18 | CI | Moderate | OPS | YZ | multiplex-pcr | Illumina | 29 | 30 | 15,000,466 | 99.94% | 55482.26 |
| YZ531 | YZ531 | 2021/8/13 | 2021/8/18 | CI | Moderate | NPS | YZ | multiplex-pcr | Illumina | 17 | 18 | 10,735,438 | 99.94% | 40623.74 |
| YZ532 | YZ532 | 2021/8/13 | 2021/8/15 | CI | Moderate | NPS | YZ | multiplex-pcr | Illumina | 16 | 18 | 1,704,304 | 99.55% | 6373.416 |
| YZ534 | YZ534 | 2021/8/13 | 2021/8/18 | CI | Moderate | NPS | YZ | multiplex-pcr | Illumina | 22 | 23 | 4,891,887 | 99.93% | 18559.2 |
| YZ535 | YZ535 | 2021/8/13 | 2021/8/15 | CI | Moderate | OPS | YZ | multiplex-pcr | Illumina | 31 | 27 | 508,379 | 99.58% | 2033.586 |
| YZ536 | YZ536 | 2021/8/14 | 2021/8/18 | CI | Moderate | NPS | YZ | multiplex-pcr | Illumina | 19 | 20 | 26,301,042 | 99.94% | 99605.61 |
| YZ537 | YZ537 | 2021/8/13 | 2021/8/18 | CI | Moderate | OPS | YZ | multiplex-pcr | Illumina | 21 | 22 | 11,139,714 | 99.94% | 42842.83 |
| YZ539 | YZ539 | 2021/8/11 | 2021/8/18 | CI | Moderate | NPS | YZ | multiplex-pcr | Illumina | 27 | 27 | 13,756,097 | 99.92% | 51334.64 |
| YZ540 | YZ540 | 2021/8/4 | 2021/8/14 | CI | Moderate | OPS | YZ | multiplex-pcr | Illumina | 23 | 24 | 790,282 | 99.82% | 3174.38 |
| YZ541 | YZ541 | 2021/8/13 | 2021/8/18 | CI | Moderate | OPS | YZ | multiplex-pcr | Illumina | 30 | 30 | 8,803,186 | 99.94% | 33754.01 |
| YZ542 | YZ542 | 2021/8/13 | 2021/8/18 | CI | Moderate | OPS | YZ | multiplex-pcr | Illumina | 19 | 20 | 6,817,804 | 99.91% | 25319.28 |
| YZ545 | YZ545 | 2021/8/13 | 2021/8/18 | CI | Moderate | NPS | YZ | multiplex-pcr | Illumina | 26 | 27 | 12,309,648 | 99.94% | 47530.96 |
| YZ546 | YZ546 | 2021/8/13 | 2021/8/15 | CI | Moderate | OPS | YZ | multiplex-pcr | Illumina | 30 | 30 | 165,921 | 99.70% | 668.936 |
| YZ547 | YZ547 | 2021/8/14 | 2021/8/18 | CI | Mild | OPS | YZ | multiplex-pcr | Illumina | 31 | 32 | 11,897,197 | 99.94% | 45605.08 |
| YZ548 | YZ548 | 2021/8/13 | 2021/8/18 | CI | Moderate | NPS | YZ | multiplex-pcr | Illumina | 18 | 19 | 11,881,278 | 99.94% | 45815.42 |
| YZ549 | YZ549 | 2021/8/10 | 2021/8/15 | CI | Moderate | NPS | YZ | multiplex-pcr | Illumina | 33 | 31 | 385,667 | 99.41% | 1536.305 |
| YZ551 | YZ551 | 2021/8/15 | 2021/8/19 | CI | Mild | NPS | YZ | multiplex-pcr | Illumina | 29 | 30 | 12,026,776 | 99.91% | 45284.34 |
| YZ552 | YZ552 | 2021/8/15 | 2021/8/18 | CI | Mild | NPS | YZ | multiplex-pcr | Illumina | 22 | 23 | 1,599,325 | 99.63% | 5909.415 |
| YZ553 | YZ553 | 2021/8/15 | 2021/8/18 | CI | Moderate | NPS | YZ | multiplex-pcr | Illumina | 27 | 28 | 12,219,751 | 99.94% | 46040.41 |
| YZ554 | YZ554 | 2021/8/5 | 2021/8/17 | II | Moderate | OPS | YZ | multiplex-pcr | Illumina | 29 | 30 | 1,866,221 | 99.92% | 7289.246 |
| YZ559 | YZ559 | 2021/8/16 | 2021/8/17 | ER | Moderate | OPS | YZ | multiplex-pcr | Illumina | 22 | 24 | 2,026,302 | 99.92% | 7550.223 |
| YZ560 | YZ560 | 2021/8/16 | 2021/8/16 | ER | Mild | OPS | YZ | multiplex-pcr | Illumina | 33 | 33 | 779,946 | 99.89% | 2702.612 |
| YZ561 | YZ561 | 2021/8/16 | 2021/8/18 | ER | Mild | OPS | YZ | multiplex-pcr | Illumina | 32 | 33 | 6,410,587 | 99.85% | 25273.11 |
| YZ562 | YZ562 | 2021/8/10 | 2021/8/18 | CI | Moderate | OPS | YZ | multiplex-pcr | Illumina | 29 | 30 | 6,127,909 | 99.85% | 24298.09 |
| YZ564 | YZ564 | 2021/8/16 | 2021/8/18 | ER | Moderate | OPS | YZ | multiplex-pcr | Illumina | 29 | 31 | 5,532,590 | 99.90% | 21918.26 |
| YZ570 | YZ570 | 2021/8/25 | 2021/8/18 | ER | Mild | OPS | YZ | multiplex-pcr | Illumina | 28 | 29 | 6,392,719 | 99.71% | 22901.38 |

a: Oropharyngeal swab. b: Nasopharyngeal Swab. c:Nanjing. d:Yangzhou. e: Guangdong. f: Yunnan. g: Thermo Fisher.
